# A distinct function of the retinoblastoma protein in the control of lipid composition identified by lipidomic profiling

**DOI:** 10.1038/oncsis.2017.51

**Published:** 2017-06-26

**Authors:** H Muranaka, A Hayashi, K Minami, S Kitajima, S Kohno, Y Nishimoto, N Nagatani, M Suzuki, L A N Kulathunga, N Sasaki, N Okada, T Matsuzaka, H Shimano, H Tada, C Takahashi

**Affiliations:** 1 Division of Oncology and Molecular Biology, Cancer Research Institute, Kanazawa University, Kanazawa, Ishikawa, Japan; 2 Exploratory Research Laboratories, Minase Research Institute, Ono Pharmaceutical Co., Ltd, Mishima, Osaka, Japan; 3 Advanced Medical Research Laboratories, Ono Pharmaceutical Co., Ltd, Tsukuba, Ibaraki, Japan; 4 Department of Medical Oncology, Dana-Farber Cancer Institute, Boston, MA, USA; 5 Department of Clinical Pharmacokinetics and Pharmacodynamics, School of Medicine, Keio University, Tokyo, Japan; 6 Department of Endocrinology and Metabolism, Faculty of Medicine, University of Tsukuba, Tsukuba, Ibaraki, Japan; 7 Oncology Research and Development Center, Minase Research Institute, Ono Pharmaceutical Co., Ltd, Mishima, Osaka, Japan

## Abstract

Here, by combining lipidomics with transcriptome analysis, we demonstrate that *Rb* depletion in mouse embryonic fibroblastss induces significant alterations in their lipid composition. We discovered that *Rb* depletion induced increase in lysophosphatidylserine, diacylglycerol (DAG), fatty acid (FA), acylcarnitine, phosphatidylcholine (PC), arachidonoyl ethanolamine, and decrease in phosphatidylglycerol, monoacylglycerol, without change in total lipid per protein levels. Analysis of the acyl chain composition of DAG, PC and phosphatidylserine revealed increase of saturated and mono-unsaturated acyl chains with specific carbon chain length. Consistently, we observed that *Rb* depletion increased the levels of fatty acids with the corresponding carbon chain length and number of carbon–carbon double bondssuch as myristic acid (14:0), palmitic acid (16:0), stearic acid (18:0) and all forms of FA 18:1. Microarray analysis revealed that *Rb* depletion induced significant upregulation of enzymes involved in elongation and desaturation of fatty acids. Among these, we found that elongation of long chain fatty acid family member 6 (Elovl6) and stearoyl-CoA desaturase 1 (Scd1) are the most robustly controlled by Rb possibly through E2F and sterol regulatory element-binding protein transcription factors. Depletion of *Elovl6* or *Scd1* significantly suppressed colony formation, sphere formation and xenograft tumor growth of *Rb*-deficient tumor cells. Suppression of self-renewal by the SCD1 inhibitor was rescued upon supplementation of the mono-unsaturated fatty acids generated by this enzyme. This study suggests a novel role for Rb in suppressing the malignant progression of tumors by controlling the lipid composition.

## Introduction

Components of the Rb pathway are functionally inactivated in the majority of human malignancies.^1^ The canonical function of Rb has been attributed to its ability to control the G1-S transition by interacting with the E2F transcription factors.^2, 3^ In addition to E2Fs, the Rb protein (pRb) interacts with a variety of transcription factors and chromatin modifiers.^4^ The heterogeneity of binding partners exceeding 300 proteins may underlie pleiotropic pRb functions exerted beyond cell cycle control including control of differentiation, apoptosis, genomic stability, senescence, angiogenesis and metastasis.^1^

More recently, emerging evidence suggests new roles for Rb in cellular metabolism, such as autophagy, glycolysis, glutaminolysis, insulin secretion, mitochondrial biogenesis, mitochondrial oxidative phosphorylation (OXPHOS), mitochondrial reactive oxygen species metabolism and mitochondrial protein translation.^5, 6, 7, 8, 9, 10^ Presumably, pRb exerts its metabolic functions largely through E2F transcription factors.

We reported that pRb controls the N-Ras activation status through protein isoprenylation (lipid modification).^11, 12^ This function of pRb appeared to be mediated dually by E2Fs and sterol regulatory element-binding proteins (SREBPs). Since SREBPs control the transcription of a variety of lipogenic genes,^13^ we hypothesized a distinct role for pRb in lipid metabolism.

Increased *de novo* lipogenesis is an early and common event in the development of cancer.^14^ Lipogenesis plays important roles in many biological processes such as generation of building blocks for membrane. Increased expression of lipogenic enzymes including ATP citrate lyase (ACLY), acetyl-CoA carboxylase A (ACACA) and fatty acid synthase (FASN) is correlated with poor prognosis in many types of cancer.^14, 15, 16, 17, 18^ In addition, numerous studies focused on the role for individual lipid metabolism enzymes in cancer.

On the other hand, given the importance of comprehending lipid composition globally, a new approach, lipidomics has emerged as a powerful tool to understand lipid metabolism.^19, 20^ Indeed, recent lipidomics studies revealed a dynamic alteration of the membrane lipid composition in human cancer tissues,^21^ and distinct lipid profiles correlated with tumor grade.^22^ Moreover, combination of molecular biology and lipidomics techniques revealed some of the mechanisms underlying deregulated lipid metabolism in cancer.^23, 24, 25, 26, 27^

Here, we integrated lipidomics studies with transcriptome analysis to uncover the role of Rb in lipid metabolism. We demonstrate that depletion of *Rb* in mouse embryonic fibroblast (MEFs) induced distinct changes in the lipid profiles. By analyzing the transcriptome in parallel, we identified a set of genes whose changes in regulation may underlie the emergence of the distinct lipidomic changes upon *Rb* depletion. Finally, we investigated the roles of these genes in malignant behaviors of cancer cells that are exerted in an Rb-dependent manner. The information described in this study indicates that Rb suppresses malignant behaviors of tumor cells at least partially by controlling their lipid composition.

## Results

### Lipid profiling of *Rb*-depleted MEFs

**Figure 1:**
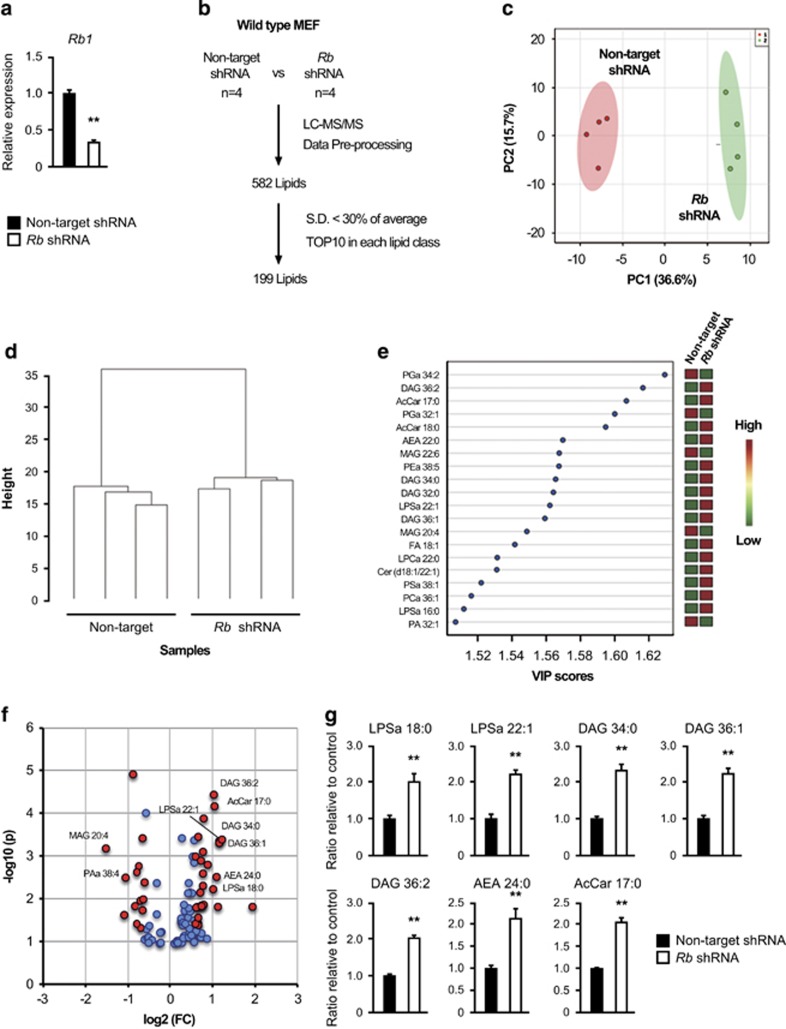
Lipidomic profiling of *Rb-*depleted MEFs. (**a**) RT–qPCR of *Rb1* in wild-type MEFs infected with lentiviruses expressing the indicated shRNAs and selected. Cells were cultured in the medium containing 1% FBS for 24 h. Data represent mean+s.d. Four batches of MEFs derived from one embryo were used per one shRNA. **P<0.01 by Student’s *t*-test. (**b**) Schematic of strategy used to identify lipids altered in *Rb-*depleted MEFs. (**c**) PCA score plot of 199 lipids (*n*=4). (**d**) Hierarchical clustering of 199 lipids (*n*=4). (**e**) Top VIP scores and heat map from PLS-DA of 199 lipids. Red and green indicate increased and decreased levels, respectively. (**f**) Volcano plot of lipids with VIP>1. Important lipids were selected with fold change threshold (*x*) and *p*-values (*t*-test) threshold (*y*). Important variables with *P*<0.05 and |FC|>1.5 were indicated with red and those with *P*<0.01 and |FC|>2 were labeled. (**g**) Levels of the indicated lipids in control MEFs (solid) and *Rb-*depleted MEFs (blank). Data represent mean+s.d. (*n*=4) **P<0.01 by Student’s *t*-test. FBS, fetal bovine serum; PCA, principal component analysis; PLS-DA, partial least square discriminant analysis.

To investigate the effect of Rb status on lipid metabolism, we employed the ultra-high performance liquid chromatography-scheduled multiple reaction monitoring lipidomics technique,^28^ and analyzed MEFs infected with lentivirus either expressing *Rb* shRNA or control shRNA. The specific effect of our *Rb* shRNA has already been validated by the provider and in a previous report from our group.^29^ Endogenous *Rb1* levels were suppressed by about 70% by *Rb* shRNA (Figure 1a). *Rb-*depleted MEFs exhibited relatively higher proliferation rate as expected (Supplementary Figure S1a). *Rb-*depleted MEFs and control MEFs were cultured in the medium containing 1% fetal bovine serum for 24 h before sample collection in one aim to minimize the effect of serum-derived lipids on the cellular lipid profile. Another aim of this treatment was to activate Rb protein in control cells. Representative mass spectra of control MEFs are shown (Supplementary Figure S1b). We analyzed four batches of wild-type MEFs from one embryo per one shRNA. After the data pre-processing, targeted lipid profiling identified a total of 582 lipids in both cell types (Figure 1b). To facilitate the initial multi- and uni-variate comparison of *Rb-*depleted and control MEFs, we reduced the number of lipids to be analyzed to 199 by removing those with a s.d.⩾30% and selecting the 10 most abundant of each lipid class (Supplementary Table S1).

Principal component analysis, which indicates variability among groups of numbers, revealed a clear difference between *Rb-*depleted and control MEFs in terms of lipid profiles (Figure 1c). The partial least square discriminant analysis score plot, which is highly related to principal component analysis, also exhibited a clear difference (Supplementary Figure S1c). Of 199 lipids, 96 showed variable importance in the projection (VIP) values (indicating importance of each variable in the variability among groups of numbers) of>1 (Supplementary Table S2). The metabolic shift after *Rb* depletion was further confirmed by hierarchical clustering analysis of 199 lipids (Figure 1d) and 25 lipids with the highest VIP scores (Supplementary Figure S1d). The heat map of the top 20 VIPs indicated the metabolites most influential in generating the variability observed; these included diacylglycerol (DAG) 36:2, acylcarnitine (AcCar) 17:0, AcCar 18:0, DAG 34:0, DAG 32:0, DAG 36:1, Cer (d18:1/22:1), phosphatidylserine (PSa) 38:1, phosphatidylcholine (PCa) 36:1 and lysophosphatidylserine (LPSa) 16:0 that are commonly composed of C16:0, C16:1, C17:0, C18:0 or C18:1 fatty acyl chains (carbon chain length: number of carbon–carbon double bonds) (Figure 1e). It is worth noting that FA 18:1 also increased in *Rb-*depleted MEFs. Next, we generated the volcano plot (indicating fold change together with significance) of the −log10 (p) versus log2 (FC) values (*Rb-*depleted versus control MEFs) of all variables with VIP>1, and visualized the important variables with *P*<0.05 and |FC|>1.5 (indicated with red) (Figure 1f). Among these, lipids with *P*<0.01 and |FC|>2 were shown in Figure 1g and Supplementary Figure S1e. The upregulated compositions contained LPSa 18:0, LPSa 22:1, DAG 34:0, DAG 36:1, DAG 36:2, ethanolamine (AEA) 24:0 and AcCar 17:0, while the downregulated ones contained PAa 38:4 and monoacylglycerol (MAG) 20:4. We detected again many of those upregulated (LPSa18:0, DAG 34:0, DAG 36:1, DAG 36:2 and AcCar 17:0) are chiefly composed of specific acyl chains including C16:0, C16:1, C17:0, C18:0 or C18:1.

### *Rb* depletion induces a shift of acyl chain composition in diacylglycerol

**Figure 2:**
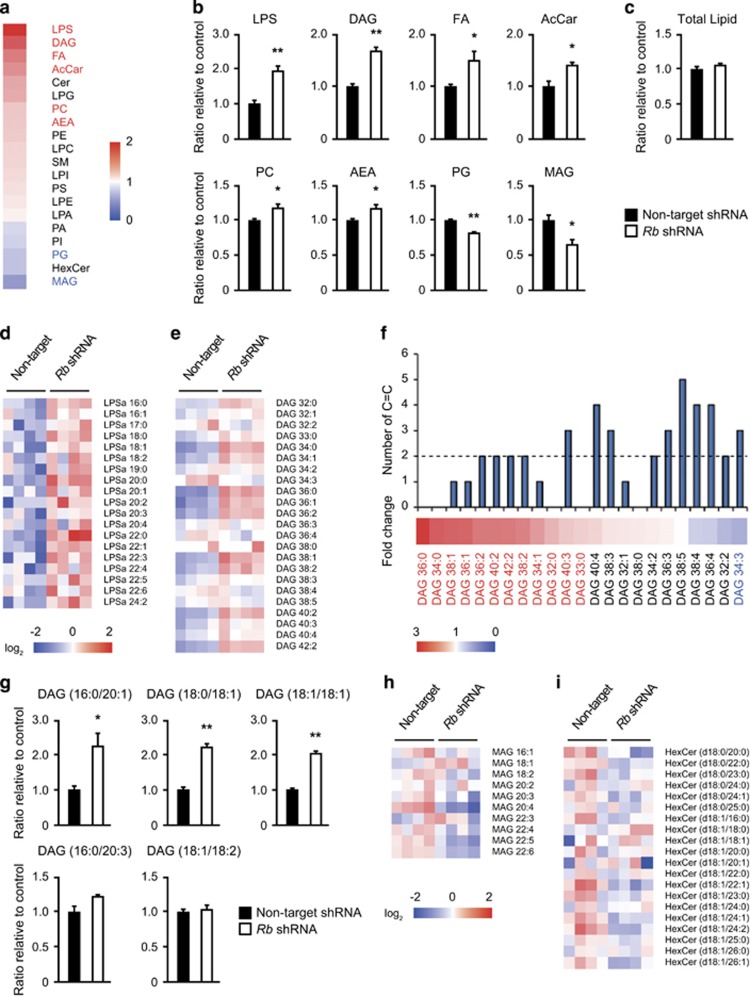
The impact of *Rb* depletion on lipid class and acyl chain composition. (**a**) Heat map of the mean fold change in each lipid class between control MEFs and *Rb-*depleted MEFs. Red, increase; white, average; blue, decrease. Names of the lipid class shown in red are significantly increased, and blue are significantly decreased in *Rb-*depleted MEFs. Four batches of MEFs derived from one embryo were used per one shRNA. *P*<0.05 by Student’s *t*-test. (**b**) Levels of the indicated lipid class in control MEFs (solid) and *Rb-*depleted MEFs (blank). Data represent mean+s.d. (*n*=4) *P<0.05, **P<0.01 by Student’s *t*-test. (**c**) The total lipid content in control MEFs (solid) and *Rb-*depleted MEFs (blank). Data represent mean+s.d. (*n*=4). (**d**, **e**) Heat map represents log 2 fold changes in the indicated two different lipid species in *Rb-*depleted MEFs as compared to control MEFs. Red, increase; white, average; blue, decrease. (**f**) Heat map of the mean fold change in DAG species in *Rb-*depleted MEFs as compared to control MEFs (lower). Red, increase; white, average; blue, decrease. Names of the lipid class shown in red are significantly increased, and blue are significantly decreased in *Rb-*depleted MEFs (*P*<0.05 by Student’s *t*-test). The number of carbon–carbon double bonds (C=C) for each lipids is shown in the bar graph (upper). (**g**) Levels of the indicated DAG species in control MEFs (solid) and *Rb-*depleted MEFs (blank). Data represent mean+s.d. (*n*=4) *P<0.05, **P<0.01 by Student’s *t*-test. (**h**, **i**) Heat map represents log 2 fold changes in the different lipid species in *Rb-*depleted MEFs as compared to control MEFs. Red, increase; white, average; blue, decrease.

In addition to the significant increase in specific LPS and DAG species, we observed marked increase in the total amount of LPS and DAG upon *Rb* depletion (Figures 2a and b). We also found significant increase in the total amount of FA, AcCar, PC and ethanolamine (Figures 2a and b). On the other hand, the amount of phosphatidylglycerol and MAG decreased significantly (Figures 2a and b). However, these changes occurred without alterations in total lipid amount per protein (Figure 2c).

We next assessed the pattern of changes in individual lipid species in each lipid class. Among lipid classes we analyzed, LPS species were almost uniformly upregulated (Figure 2d). However, most of the upregulated DAG species had zero, one or two carbon-carbon double bonds, while those with more than three double bonds had no change (Figure 2e). This pattern appeared to be obvious when all the species were re-aligned according to the mean fold change in each DAG species between *Rb-*depleted and control MEFs (Figure 2f). VIP score analysis exhibited a similar trend (Supplementary Figure S2a). Hierarchial clustering analysis of DAG species indicated that independent clusters might reflect the difference in the number of carbon–carbon double bonds (Supplementary Figure S2b). These data support the idea that *Rb* depletion increased a series of DAG species of particular number of carbon–carbon double bonds, and the change in these DAG species accounts for the increase in the total amount of DAG.

Next, we assessed the acyl chain composition of each DAG species according to the data acquired by linear ion trap data-dependent MS/MS (Supplementary Table S3). We observed an increase in DAG 36:1 (16:0/20:1 or 18:0/18:1) and DAG 36:2 (18:1/18:1) in *Rb-*depleted MEFs (Figure 2g). In contrast, DAG 36:3 (16:0/20:3 or 18:1/18:2) was not influenced by *Rb* depletion (Figure 2g). These data indicate that *Rb* depletion increased particular DAG species composed of C16:0, C18:0 or C18:1 acyl chains. This idea was further supported by the data of the other DAG species with different carbon length (Supplementary Table S3).

We then assessed whether any changes in acyl chain composition occur in other lipid class especially DAG-derived lipid metabolites upon *Rb* depletion. We found that among glycerophospholipids derived from DAG, PC and phosphatidylserine showed a pattern similar to that was seen in DAG (Supplementary Figures S3a and b). Other lipids, however, showed different patterns of changes (Supplementary Figures S3c–h).

The amount of total MAG was downregulated (Figure 2h). However, we noticed that MAG 18:1 in particular was upregulated. Similarly, hexosyl-ceramide (HexCer) was mostly downregulated but HexCer (d18:1/18:0) was upregulated (Figure 2i). These findings suggest that the distinct pattern of changes in acyl chain composition induced upon *Rb* depletion is shared by multiple lipid species regardless of change in total amount.

### *Rb* depletion induces a distinct qualitative change in fatty acids

**Figure 3:**
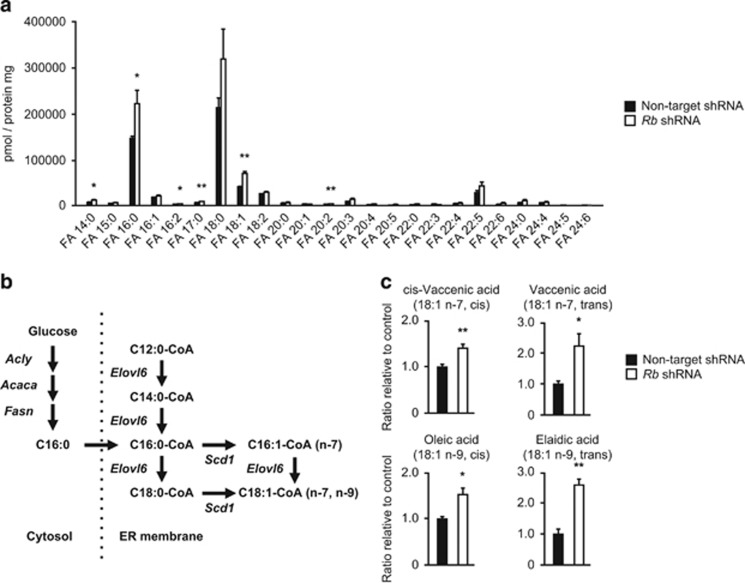
Rb has significant impact on fatty acid composition. (**a**) Levels of the indicated fatty acids in control MEFs (solid) and *Rb-*depleted MEFs (blank). Data represent mean+s.d. Four batches of MEFs derived from one embryo were used per one shRNA. *P<0.05, **P<0.01 by Student’s *t*-test. (**b**) Diagram of *de novo* synthesis of MUFAs. (**c**) Levels of the indicated FA 18:1 in control MEFs (solid) and *Rb-*depleted MEFs (blank). Data represent mean+s.d. (*n*=4) *P<0.05, **P<0.01 by Student’s *t*-test. MUFA, mono-unsaturated fatty acid.

We sought a possible mechanism underlying the distinct qualitative changes in multiple lipid species that appeared upon *Rb* depletion. Since the pattern that appeared in multiple lipid species entails an increase in the levels of fatty acyl chains with a specific number of carbons and carbon–carbon double bonds, we focused on the fatty acids composition. With respect to the robust quantitative variability in fatty acids with different carbon length and degrees of unsaturation, we compared the absolute concentrations of each species. We found that the absolute amount of FA 16:0 and FA 18:1 significantly increased (Figure 3a). We also observed an increase in FA 18:0 but with poor statistical significance in this assessment. Among fatty acids present to a smaller amount, we observed significant increase in FA 14:0, FA 16:2, FA 17:0 and FA 20:2 upon *Rb* depletion (Figure 3a). The heat map of the VIP projection analysis showed the highest score for FA 18:1 (Supplementary Figure S4a). We then quantified all forms of FA 18:1 (Figure 3b). Cis-vaccenic acid (18:1 n-7, cis), vaccenic acid (18:1 n-7, trans), oleic acid (18:1 n-9, cis) and elaidic acid (18:1 n-9, trans) were all significantly upregulated upon *Rb* depletion (Figure 3c).

Since glycerophospholipids are formed by the association of glycerols and fatty acids, we thought that an increase in the levels of particular glycerophospholipids might be explained by higher levels of particular fatty acids. We conducted a correlation coefficient analysis for DAG 34:1, DAG 36:1 and DAG 36:2 that were significantly increased by *Rb* depletion. We found that an increase in the levels of FA 18:1 has a strong correlation with the increase in the levels of these DAGs (Supplementary Figure S4b). In addition, the correlation coefficient analysis indicated a strong positive correlation between FA 18:1 and several lipid species possibly composed of FA 18:1 including DAG 36:1, DAG 36:2 and DAG 34:1 (Supplementary Figure S4c). All these findings suggest that an increase in the levels of particular fatty acids upon *Rb* depletion is strongly correlated with an increase of glycerophospholipids with specific fatty acyl chains.

The pathway leading to FA 18:1 synthesis includes FA 14:0, FA 16:0 and FA 18:0; the elongation and desaturation of all these lipids are performed by the two enzymes Elovl6 and Scd1 (Figure 3b). The findings of our lipidomic analyses indicated that Elovl6 and Scd1 might be upregulated by *Rb* depletion.

### Rb regulates transcription of *Elovl6* and *Scd1*

**Figure 4:**
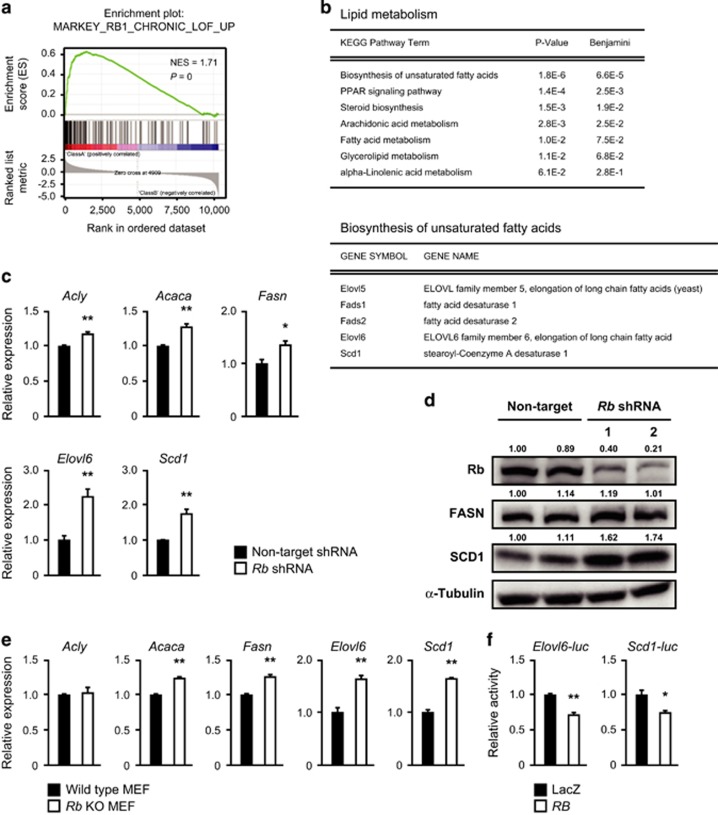
Rb regulates *Elovl6* and *Scd1* expression. (**a**) GSEA result for *RB1* target gene set in *Rb-*depleted MEFs versus control MEFs. (**b**) GO analysis of upregulated genes in lipid metabolism in *Rb-*depleted MEFs (upper). Upregulated genes in biosynthesis of unsaturated fatty acids are shown (lower). (**c**) RT–qPCR of the indicated genes in wild-type MEFs infected with lentiviruses expressing the indicated shRNAs and selected. Cells were cultured in the medium containing 1% FBS for 24 h. Data represent mean+s.d. Four batches of MEFs derived from one embryo were used per one shRNA. *P<0.05, **P<0.01 by Student’s *t*-test. (**d**) Immunoblotting (IB) of the indicated proteins in wild-type MEFs transduced with the indicated shRNA and cultured in the medium containing 1% FBS for 24 h. Band intensities were quantified by ImageJ software and indicated. (**e**) RT–qPCR of the indicated genes in *Rb*^+/+^ MEFs and *Rb*^−/−^ MEFs. Cells were cultured in the medium containing 1% FBS for 24 h. Data represent mean+s.d. Three batches of MEFs derived from each embryo were used per one shRNA. *P<0.05, **P<0.01 by Student’s *t*-test. (**f**) Luciferase activity in wild-type MEFs transfected with pGL3 containing the indicated luciferase reporters together with pCMV-β-gal and pCMV vectors expressing the indicated proteins. Results are normalized to β-gal activity. Data represent mean+s.d. (*n*=3) *P<0.05, **P<0.01 by Student’s *t*-test. FBS, fetal bovine serum; GO, Gene Ontology; GSEA, Gene Set Enrichment Analysis.

To specifically determine the role of Rb in lipid metabolism, we conducted a microarray analysis of *Rb-*depleted MEFs (*Rb* shRNA1) (Supplementary Table S4). The signature obtained from the comparison of *Rb-*depleted and control MEFs was significantly similar to an RB inactivation signature determined in a previous work^30^ when assessed by Gene Set Enrichment Analysis, suggesting that *Rb* depletion was successful (Figure 4a). As expected, Gene Ontology analysis of the signature contained Kyoto Encyclopedia of Genes and Genomes pathway terms related to cell cycle and DNA synthesis with high significance (Supplementary Table S5). We then selected gene sets related to lipid metabolism from our RB inactivation signature, and applied them to Gene Ontology analysis again (Figure 4b and Supplementary Table S6). We consequently found that a series of genes involved in the biosynthesis of unsaturated fatty acids including *Elovl6* and *Scd1* were significantly upregulated following *Rb* depletion (Figure 4b). We confirmed this finding by conducting reverse transcription and real-time quantitative PCR (RT–qPCR); *Elovl6* and *Scd1* were robustly upregulated in *Rb-*depleted MEFs (Figure 4c). The increase in FA 16:0 can be explained by the increased *de novo* fatty acid synthesis. Thus in addition to *Elovl6* and *Scd1*, we conducted an RT–qPCR of *Acly*, *Acaca* and *Fasn* (Figure 3b). All these enzymes were slightly but significantly increased upon *Rb* depletion (Figure 4c and Supplementary Table S7). Immunoblotting partially confirmed our RT–qPCR results (Figure 4d). Additionally, we analyzed MEFs in which *Rb* is depleted using another shRNA (*Rb* shRNA2). *Rb* shRNA2 downregulated *Rb1* expression to a lesser extent than shRNA1 did. This resulted in a weaker upregulation of *Elovl6* and *Scd1*, confirming that these genes were not upregulated because of off-target effects of shRNAs (Supplementary Figure S5a). We also analyzed MEFs derived from another pair of *Rb*^−/−^ and *Rb*^+/+^ mouse embryo. The results were highly consistent with the analysis using *Rb-*depleted MEFs (Figure 4e and Supplementary Figure S5b). Moreover, analysis of our and other’s previous microarray data acquired in *Rb*-deficient or *RB* overexpression contexts identified *Elovl6* and *Scd1* as likely targets of Rb (Supplementary Table S8). Finally, luciferase reporter assay revealed that RB significantly suppressed Elovl6 and Scd1 promoter activity (Figure 4f). Taken together, these findings suggest that Rb status affects the expression of several genes involved in fatty acid synthesis, elongation and desaturation; among them, *Elovl6* and *Scd1* are the ones most robustly affected.

### Possible involvement of E2Fs and SREBPs in the transcriptional control of *Elovl6* and *Scd1*

**Figure 5:**
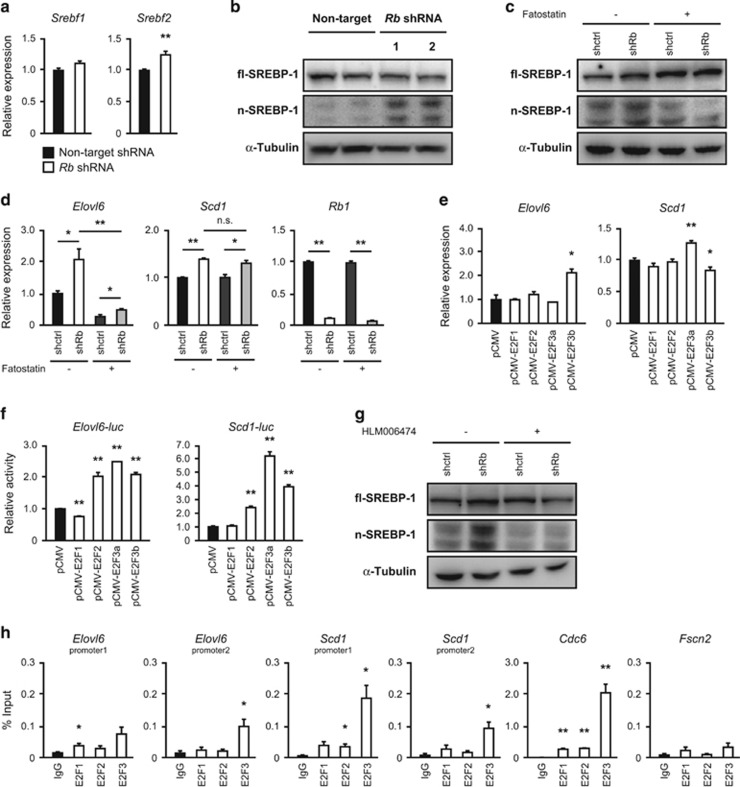
The mechanism whereby Rb regulates *Elovl6* and *Scd1* gene expression. (**a**) RT–qPCR of the indicated genes in wild-type MEFs infected with lentiviruses expressing the indicated shRNAs and selected. Cells were cultured in the medium containing 1% FBS for 24 h. Data represent mean+s.d. Four batches of MEFs derived from one embryo were used per one shRNA. *P<0.05, **P<0.01 by Student’s *t*-test. (**b**) IB of the indicated proteins in wild-type MEFs transduced with the indicated shRNA and cultured in the medium containing 1% FBS for 24 h. Full-length of SREBP-1 (fl) and nuclear SREBP-1 (n) were separated. (**c**) IB of the indicated proteins in wild-type MEFs transduced with the indicated shRNA and cultured in the medium containing 1% FBS for 24 h with or without SREBP inhibitor fatostatin (20 μM) for last 6 h. (**d**) RT–qPCR of the indicated genes in wild-type MEFs infected with lentiviruses expressing the indicated shRNAs and selected. Cells were cultured in the medium containing 1% FBS for 24 h with or without fatostatin (20 μM) for last 6 h. Data represent mean+s.d. Three batches of MEFs derived from one embryo were used per one shRNA. *P<0.05, **P<0.01 by Student’s *t*-test. (**e**) RT–qPCR of the indicated genes in *Rb*^−/−^ MEFs transfected with pCMV vectors expressing the indicated proteins. Data represent mean+s.d. (*n*=3) *P<0.05, **P<0.01 by Student’s *t*-test. (**f**) Luciferase activity in HepG2 cells transfected with pGL3 containing the indicated luciferase reporters together with pCMV-β-gal and pCMV vectors expressing the indicated proteins. Results are normalized to β-gal activity. Data represent mean+s.d. (*n*=3) *P<0.05, **P<0.01 by Student’s *t*-test. (**g**) IB of the indicated proteins in wild-type MEFs transduced with the indicated shRNA and cultured in the medium containing 1% FBS for 24 h with or without HLM006474 (30 μM). (**h**) Association of E2F1, E2F2 and E2F3 with the promoters of the mouse *Elovl6*, *Scd1*, *Cdc6* (positive control) and *Fscn2* (negative control) was assessed by ChIP in *Rb*^−/−^ MEFs. Input or eluted chromatin was subjected to real-time PCR analysis using promoter-specific primers. Data are represented as the % input of the immunoprecipitated chromatin for each gene from three separate chromatin preparations. Data represent mean+s.d. (*n*=3) *P<0.05, **P<0.01 by Student’s *t*-test. FBS, fetal bovine serum.

We next addressed the mechanism by which Rb controls the expression of *Elovl6* and *Scd1*. Since we had previously linked Rb to N-Ras maturation through SREBP-dependent regulation of isoprenylation-related genes,^12^ we assessed the status of SREBP-1 and 2 in *Rb-*depleted MEFs. Unlike previously investigated C cells, *Rb* depletion in MEFs only slightly increased the transcription of *Srebf2*, but not *Srebf1* (Figure 5a). When we compared *Rb*^−/−^ MEFs to littermate *Rb*^+/+^ MEFs, *Srebf1* expression was increased in cells lacking *Rb* (Supplementary Figure S5c). Surprisingly, we however observed that the nuclear form of SREBP-1 was significantly enhanced both in *Rb-*depleted MEFs and in *Rb*^−/−^ MEFs compared to their control cells (Figure 5b and Supplementary Figure S5d). Among the regulators of SREBP nuclear translocation, *Insig2* was downregulated both in *Rb-*depleted MEFs and in *Rb*^−/−^ MEFs compared to control cells (Supplementary Figures S5e and f). Furthermore, nuclear translocation of SREBP-1 induced by *Rb* depletion was antagonized by fatostatin treatment, which inhibits nuclear translocation of SREBPs (Figure 5c). Thus, although the effects of knockdown and knockout were somewhat different, we conclude that *Rb* depletion in MEFs promotes nuclear translocation of SREBP-1. Additionally, fatostatin significantly antagonized *Rb* depletion-induced upregulation of *Elovl6* but not *Scd1* (Figure 5d). Collectively, these results indicate that *Rb* inactivation increases *Elovl6* gene expression through SREBP-1 activation.

Since we identified a number of RB partner E2F-binding consensus sequences in addition to sterol regulatory elements in the promoter region of these genes both in mice and human (Supplementary Figure S6a and Supplementary Table S9) and Gene Set Enrichment Analysis indicated that *Rb* depletion in MEFs significantly induced upregulation of E2F targets (Supplementary Figure S6d), we addressed the possibility that *Elovl6* and/or *Scd1* are directly regulated by E2Fs. This idea is supported by the finding that *Rb* depletion can induce both *Elovl6* and *Scd1* in *Srebf1*^−/−^ MEFs (Supplementary Figure S6b). In addition, depletion of *Srebf2* could not antagonize *Rb* depletion-induced upregulation of *Elovl6* and *Scd1* (Supplementary Figure S6c). Overexpression of individual transactivating E2F family members indicated the possibility that both *Elovl6* and *Scd1* genes are sensitive to overexpression of some of the RB partner E2Fs (Figure 5e). Furthermore, E2F2 and E2F3 enhanced the luciferase activity of both *Elovl6* and *Scd1* (Figure 5f). Importantly, we discovered that nuclear translocation of SREBP-1 induced by *Rb* depletion was significantly antagonized by an E2F inhibitor HLM006474, indicating that SREBP-1 nuclear translocation depends on E2Fs (Figure 5g). We then performed chromatin immunoprecipitation (ChIP) assay to assess direct regulation of *Elovl6* and *Scd1* gene by E2Fs. We found that E2F3 occupies the proximal promoter of both *Elovl6* and *Scd1* with a statistical significance (Figure 5h). We also found that E2F1 occupies *Elovl6* promoter and E2F2 occupies *Scd1* promoter to a lesser extent, but with a statistical significance. ChIP assay of E2Fs binding to a known target *Cdc6* promoter (positive control) showed all of E2F1, E2F2 and E2F3 occupy it. In addition, none of them interacted with *Fscn2* gene promoter (negative control). These results are consistent with our other data, indicating that E2F3 had a strongest effect on *Elovl6* and *Scd1* gene expression (Figures 5e and f). Taken together, these findings suggest the involvement of E2Fs in the transcriptional control of *Elovl6* and *Scd1*.

### *Elovl6* and *Scd1* depletion suppresses the malignant phenotype of *Rb*-deficient cells

**Figure 6:**
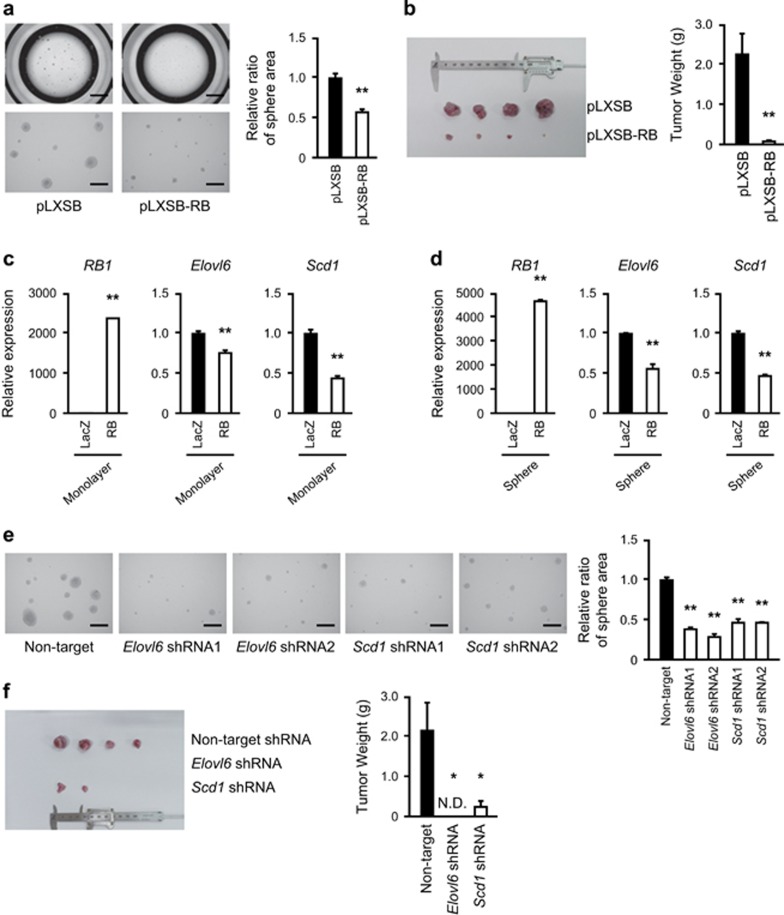
Effects of inhibition of *de novo* MUFAs synthesis on sphere formation induced by Rb inactivation. (**a**) Sphere assay of RN6 cells infected with pLXSB vectors expressing the indicated proteins and selected. 2 × 10^3^ cells were seeded and sphere images were acquired after 7 days. Scale bars, 1,500 μm (top) and 300 μm (bottom). Data represent mean+s.d. (*n*=4). *P<0.05, **P<0.01 by Student’s *t*-test. (**b**) Tumors developed in KSN/Slc mice transplanted with RN6 cells transduced with the indicated expression vectors and selected. 1 × 10^4^ cells were injected subcutaneously into male KSN/Slc mice. Tumors were weighed 46 days after injection. Data represent mean+s.d. (*n*=4) *P<0.05, **P<0.01 by Student’s *t*-test. (**c**, **d**) RT–qPCR of the indicated genes in RN6 cells transduced with the indicated proteins and cultured under the indicated conditions. Data represent mean+s.d. (*n*=3–5). *P<0.05, **P<0.01 by Student’s *t*-test. (**e**) Sphere assay of RN6 cells infected with lentiviruses expressing the indicated shRNAs and selected. 2 × 10^3^ cells were seeded and sphere images were acquired after 7 days. Scale bars, 300 μm. Data represent mean+s.d. (*n*=4). *P<0.05, **P<0.01 by Student’s *t*-test. (**f**) Tumors developed in KSN/Slc mice transplanted with RN6 cells infected with lentiviruses expressing the indicated shRNAs and selected. 1 × 10^4^ cells were injected subcutaneously into male KSN/Slc mice. Tumors were weighed 46 days after injection. Data represent mean+s.d. (*n*=4) *P<0.05, **P<0.01 by Student’s *t*-test. MUFA, mono-unsaturated fatty acid.

Given the genetic interactions between *Rb* and *Elovl6,* and between *Rb* and *Scd1*, we finally examined whether Elovl6 and Scd1 contribute to the malignant behavior of *Rb*-deficient cells. To this end, we employed RN6 cells derived from *Rb*^−/−^; *N-ras*^−/−^ MEFs that spontaneously acquired a loss of function mutation in *Trp53*.^29^ This line exhibits a carcinogenic phenotype represented by an enhanced ability to form spheres, colonies and tumors in an Rb-dependent manner (Figures 6a and b and Supplementary Figures S7a). Overexpression of RB significantly suppressed these phenotypes. We found that *Elovl6* and *Scd1* expression was downregulated upon overexpression of RB in RN6 cells under both monolayer and sphere culture conditions (Figures 6c and d).

It was previously reported that RN6 cells cultured under sphere-forming conditions showed increased expression of *Nanog* and *Sox2* when compared to cells grown under monolayer culture condition.^29^ We observed a significant upregulation of *Elovl6* and *Scd1* in sphere cultures, implicating that these enzymes might positively contribute to the self-renewal stimulated by *Rb* deficiency (Supplementary Figures S7b). To test this hypothesis, we depleted either *Elovl6* or *Scd1* in RN6 cells (Supplementary Figure S7c). This resulted in significant suppression of sphere formation (Figure 6e). Moreover, depletion of either *Elovl6* or *Scd1* in RN6 cells resulted in significant attenuation of colony formation and tumor initiation in immune-compromised mice (Supplementary Figures S7d and Figure 6f). A chemical inhibitor of SCD1 (MF-438) almost completely suppressed sphere formation by RN6 cells in consistent with the knockdown results (Supplementary Figure 7e). Moreover, the effect of this agent was rescued by supplementation of bovine serum albumin-conjugated mono-unsaturated fatty acids (FA 16:1 or FA 18:1) but not by supplementation of saturated fatty acids (FA 16:0 or FA 18:0). These findings indicate that desaturation of fatty acids may contribute to the malignant behavior of cancer cells induced by *Rb* loss.

### Correlation of *RB* mutation with *ELOVL6* and *SCD1* gene expression in human cancer patients

To investigate whether the regulation of *Elovl6* and *Scd1* gene expression by Rb is applicable to human cancer patients, we analyzed cBioPortal database. Remarkably, *ELOVL6* and *SCD1* exhibited significantly higher expression levels in mutant *RB* tumors compared to those bearing wild-type *RB* in breast cancer and ovarian cancer patients (Supplementary Figure S8).

## Discussion

RB inactivation during tumor progression is correlated with the gain of malignant features by cancer cells such as epithelial–mesenchymal transition, undifferentiated phenotypes, angiogenesis, metastasis and therapy resistance.^1^ Gene products or metabolites that quantitatively or qualitatively change in correlation with the RB status in these phenomena might contain novel therapeutic targets for cancer.^5, 6, 7, 8, 9, 10^ We therefore have been determining the Rb inactivation signatures of mRNA, microRNA and metabolites in various cellular contexts.^31, 32^ In the current study, by employing an advanced lipidomics technique, we succeeded in determining a lipidomic signature associated with Rb inactivation.

Recently, lipidomics techniques have been applied to cancer studies by an increasing number of researchers.^21, 22, 23, 24, 25, 26, 27^ The mechanisms by which the activation of oncogenes or the inactivation of tumor suppressor genes promote deregulated lipid metabolism in tumor cells are becoming clearer.^33, 34, 35, 36^ In this study, we employed an advanced lipidomics method called scheduled multiple reaction monitoring that can simultaneously detect and quantify thousands of fatty acid metabolites without deterioration of data quality. This is enabled by the MS/MS scanning technique for determining choromatographic elution peak of each target lipid molecule that can be achieved within a short time (2~3 min).

Our results demonstrated that changes in the Rb status gives rise to a profound impact on the lipidomic signature. *Rb* depletion in MEFs caused a significant increase in LPS, DAG, FA, AcCar, PC and ethanolamine, and significant decrease in phosphatidylglycerol and MAG, without changes in the total lipid amount per protein. We observed a unique change in acyl chain composition in multiple lipid classes including DAG, PC and phosphatidylserine. This change can be at least partially explained by the changes in the FA composition, since the profile of acyl chains in these lipid classes are similar to FA composition; we observed increased levels of myristic acid (14:0), palmitic acid (16:0), stearic acid (18:0) and all forms of FA 18:1. As membrane phospholipids are constantly synthesized and remodeled, it is reasonable that the changes in the FA composition affect the acyl chain composition of these lipid classes. Furthermore, increased FA production and changes in FA composition can be explained by the accelerated elongation and desaturation of fatty acids because of an elevated transcription of *Elovl6* and *Scd1* induced by *Rb* depletion. Inhibition of either ELOVL6 or SCD1 was in fact reported to largely affect the composition of fatty acids and phospholipids in cancer cells.^27, 37^ Therefore, transcriptional regulation of *Elovl6* and *Scd1* might explain the Rb function in the control of lipid metabolism to a large extent.

We observed that nuclear translocation of SREBP-1 is enhanced by *Rb* depletion in MEFs in an E2F-dependent manner. The promoter region of mouse *Elovl6* and *Scd1* possess both E2F and SREBP-binding sequences, and indeed ChIP assay demonstrated the direct involvement of E2Fs in the transcriptional regulation of *Elovl6* and *Scd1*. These findings indicate an intimate coordination of E2Fs and SREBPs in the control of lipid metabolism by RB.

Increase in DAG might also be explained by elevated *Lpin1* transcription (Supplementary Table S4), whose product synthesizes DAG from PA.^38^ In addition to *Elovl6*, *Scd1* and *Lpin1*, our microarray analysis of *Rb-*depleted MEFs detected changes in the expression levels of many genes related to lipid metabolism; the biological significance will be investigated in the future. As of now, it would be useful to assess the effect of upregulated DAG on cellular signaling, as it functions as a second messenger.^39^ Changes in the acyl chain composition of membrane lipids such as PC might affect the topology or activity of membrane proteins by altering local membrane structure or thickness.^40, 41, 42^ In future we will study whether the Rb status can affect these features through the control of lipid metabolism.

There are a few previous studies that connected Rb to lipid metabolism. Our group reported that Rb regulates many enzymes implicated in the mevalonate and lipid synthesis pathways through E2Fs and SREBPs.^12^ In addition, it was shown that E2F1 participates in the control of fatty acid metabolism in Sonic hedgehog (Shh)-driven mice medulloblastoma.^43^ This work demonstrated that Sonic hedgehog signaling triggers *de novo* fatty acid synthesis through the Sonic hedgehog-Rb/E2F1-FASN axis while suppressing fatty acid oxidation. Recently, another group suggested that E2F1 regulates lipogenesis thereby contributing to mouse liver steatosis.^44^ The same body of work demonstrated that loss of *E2f1* suppresses the expression of lipogenic genes including *Acaca*, *Fasn*, *Scd1*, *Srebp1c* and *Chrebp* in mouse livers and primary hepatocytes, supporting our current work.

We finally demonstrated that inhibition of Elovl6 and Scd1 suppresses malignant phenotypes induced by Rb inactivation. Since RB is frequently inactivated during tumor progression, these two enzymes can be promising therapeutic targets. Regarding SCD1, numerous studies already reported its clinical relevance. Inhibition of SCD1 in cancer cells induces apoptosis by inducing ER stress or cytochrome c release.^27, 45, 46^ In other contexts, inhibition of SCD1 causes growth suppression by attenuating EGFR phosphorylation or palmitoylation of WNT3A.^47, 48^ ELOVL6 is overexpressed in several cancers including nonalcoholic steatohepatitis-related hepatocellular carcinoma,^49, 50^ squamous cell carcinoma^37^ and breast cancer,^51^ although the mechanism of its role in cancer cells is not yet clear. Mice homozygously deficient for these genes are viable.^52, 53, 54^ Hence, inhibitors of these enzymes might exhibit tumor-specific effects. Taken together, our current work highlights a critical role for Rb in the control of lipid metabolism and, by a comprehensive approach combining lipidomics and transcriptomics, identifies lipid metabolism enzymes as possible therapeutic targets.

## Materials and methods

### Animals

*Rb*^+/−^ mice were obtained from Dr T. Jacks.^55^ *Srebf1*^−/−^ mice were obtained from Dr H. Shimano.^56^ All animals were handled in accordance with the guidelines of Kanazawa University.

### Generation and culture of MEFs

MEFs were isolated from littermate wild type, *Rb*^−/−^ embryos and *Srebf1*^−/−^ embryos as described previously,^57^ and maintained in α modified Eagle’s medium (MEMα, #135-15175, Wako, Osaka, Japan) supplemented with 10% fetal bovine serum and 1% phosphatidylserine.

### Virus production and transduction

Lentivirus or retrovirus production and transduction were performed as described previously.^12, 29^ MISSION TRC shRNA target sets used in this study were purchased from Sigma-Aldrich (St Louis, MO, USA) and shown in Supplementary Materials and Methods. pLXSB, pLXSB-RB, pLenti6.3/V5-LacZ and pLenti6.3/V5-HA-RB were described previously.^12, 29^

### Lipidomics

MEFs were grown in MEMα with 1% fetal bovine serum for 24 h, trypsinized, and collected in complete culture medium. The collected cells were washed two times with PBS, isolated by centrifugation, and stored at −80 °C before lipid extraction. Next, samples were mixed with 300 μl methanol containing 1% acetic acid and sonicated on ice, followed by the addition of 2 ml of a 1:2:2 mixture of chloroform:methanol:ethanol containing the internal standards and 100 μl of a 2:1:1:1 mixture of chloroform:methanol:ethanol:acetic acid. The mixture was vortexed at room temperature, centrifuged for 10 min at 4 °C; the organic layer was collected, evaporated under reduced pressure and dissolved in 280 μl reconstitution solution of which 4 μl was analyzed by LC-MS/MS. After the data pre-processing, lipids with s.d.⩾30% were removed and top 10 lipids with highest quantity in each lipid class were selected. Finally 199 lipids were used for further analysis. Principal component analysis, partial least square discriminant analysis, hierarchical clustering (dendrogram and heat map) and correlation analysis were performed by MetaboAnalyst 3.0 (http://www.metaboanalyst.ca/).^58^ For more information about LC-MS/MS analysis, see Supplementary Materials and Methods.

### RT–qPCR

Total RNA extraction, RT and qPCR were performed as described previously.^29^ TaqMan probes used in this study are shown in Supplementary Materials and Methods. The mRNA levels were normalized to *Actb* or *Hprt* mRNA levels.

### Immunoblotting

Immunoblotting of whole-cell lysates and nuclear fraction was performed as described previously.^57^ Antibodies used in this study are shown in Supplementary Materials and Methods. Quantification of the band intensity was performed by ImageJ Version 1.45s software.

### Luciferase assay

MEFs and HepG2 cells were co-transfected with pGL3-mouse Elovl6 or pGL3-mouse Scd1 (gifted by Dr Shimano),^59^ pCMV-β-gal and the indicated vectors using Lipofectamine 3000 (L3000-015, Invitrogen, Carlsbad, CA, USA) according to the manufacturer’s instructions. Luciferase and β-gal activities were determined as described previously.^11^ Luciferase activities were then normalized by the β-gal activities. pCMV, pCMV-E2F1 (#24225), pCMV-E2F2 (#24226), pCMV-E2F3a (#37970) and pCMV-E2F3b (#37975) were purchased from Addgene (Cambridge, MA, USA).

### Cross-linked ChIP

ChIP experiments using MEFs were performed as previously described^60^ with some modifications. For more information, see Supplementary Materials and Methods.

### Sphere formation assay

RN6 cells derived from *Rb*^−/−^; *N-ras*^−/−^ MEFs that spontaneously acquired a loss of function mutation in *Trp53* were used.^29^ This line exhibits a carcinogenic phenotype represented by an enhanced ability to form colonies and tumors in an Rb-dependent manner. Sphere formation assay was carried out as previously described.^29^ Where indicated, sphere cultures were supplemented with bovine serum albumin or 10 μM bovine serum albumin-conjugated fatty acids in the presence of dimethylsulphoxide or 10 μM MF-438. Free fatty acids were conjugated with fatty acid-free bovine serum albumin using the method described previously.^61^ After 7 days of culture, sphere images were acquired using a BZ-8000 microscope (Keyence, Osaka, Japan) and sphere area was analyzed using BZ-II Analyzer (Keyence).

### Colony formation assay

1 × 10^3^ cells were seeded into 60 mm dishes. After 10 days of culture, colonies were stained with Giemsa solution (#1.09204.0100, MERCK, Darmstadt, Germany) and positive area for Giemsa staining was quantified by ImageJ Version 1.45s software.

### Xenograft assay

1 × 10^4^ cells were suspended in 50 μl MEMα and mixed with the same volume of Matrigel (#354234, CORNING, Corning, NY, USA). Mixtures were injected subcutaneously into male KSN/Slc mice (Japan SLC, Inc., Shizuoka, Japan). Tumors were weighed at 46 days after injection.

### Statistical analysis

Statistical significance for the relative lipids amount, mRNA expression and area of spheres, colonies were determined using unpaired Student’s *t*-test.

For more information, see Supplementary Materials and Methods.

## Supplementary Material

Supplementary Table S5, S7-S10

Supplementary Figure Legends and Tabels
