# Supplementary material for: A distinct function of the retinoblastoma protein in the control of lipid composition identified by lipidomic profiling
Source: Oncogenesis. 2017 Jun 26;6(6):e350–. doi: 10.1038/oncsis.2017.51 (PMC5519198; doi:10.1038/oncsis.2017.51)
Supplement: Supplementary Figures [file oncsis201751x1.pdf]

Muranaka et al, Supplementary Figure S1

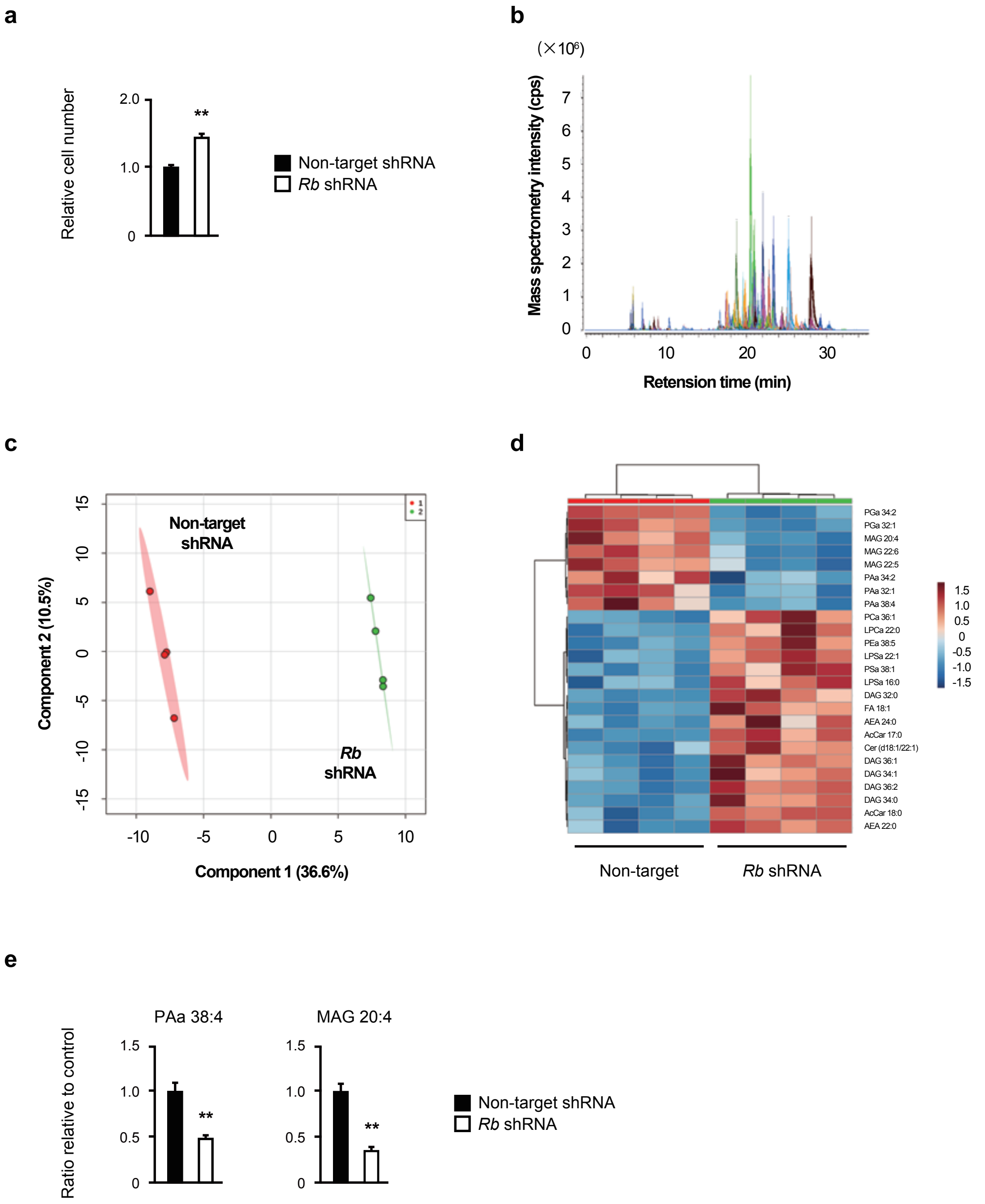

Muranaka et al, Supplementary Figure S2

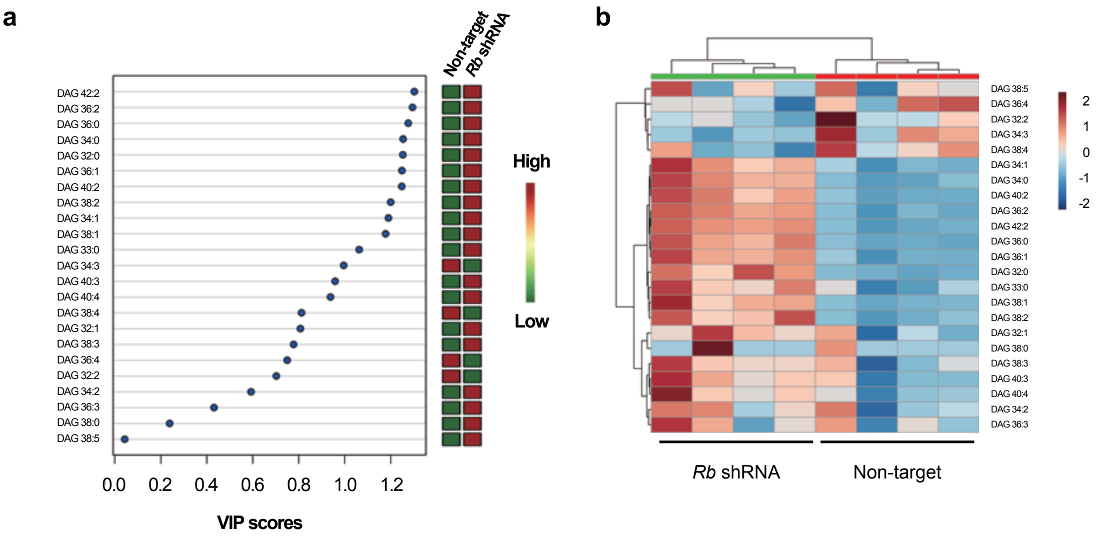

Muranaka et al, Supplementary Figure S3

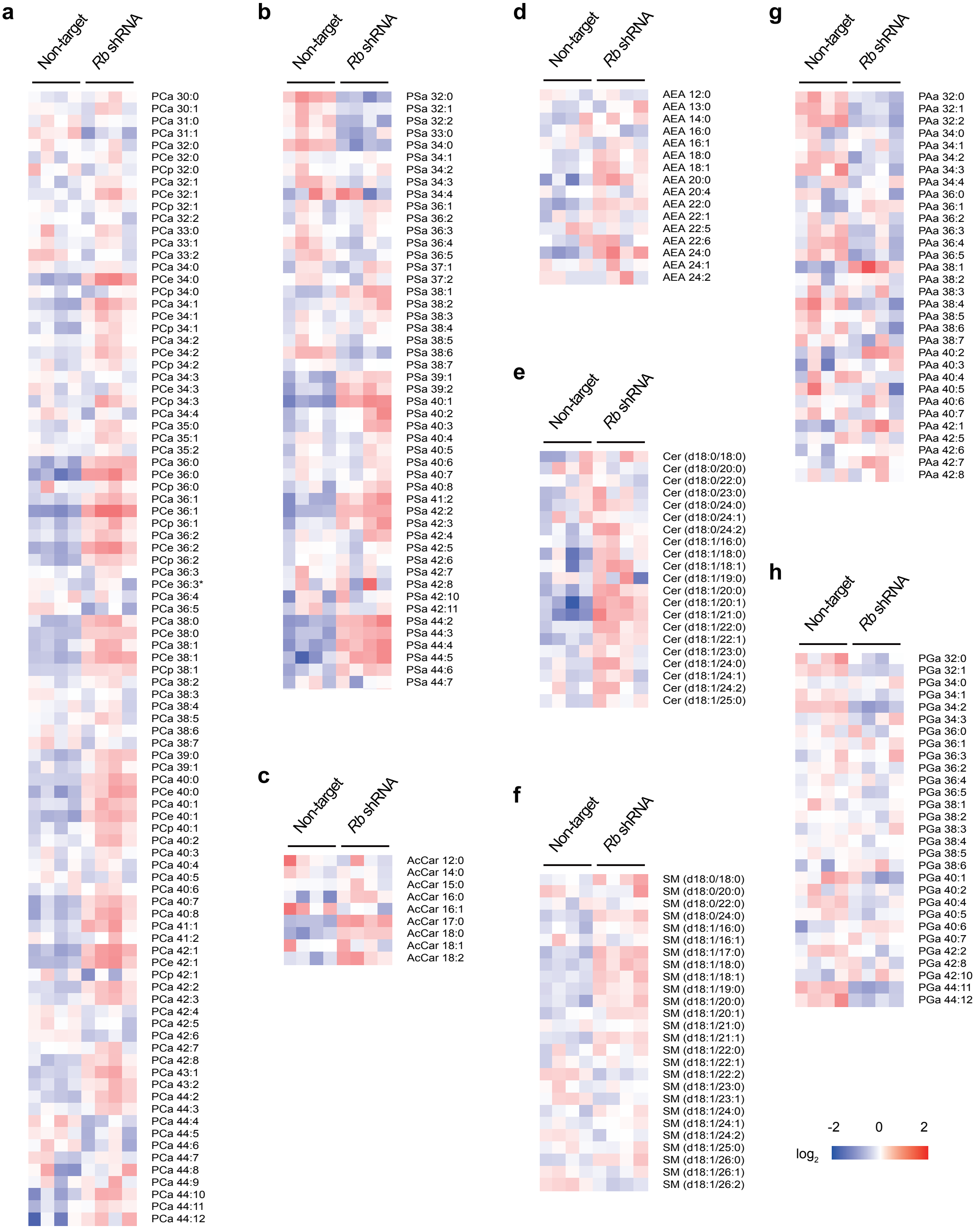

Muranaka et al, Supplementary Figure S4

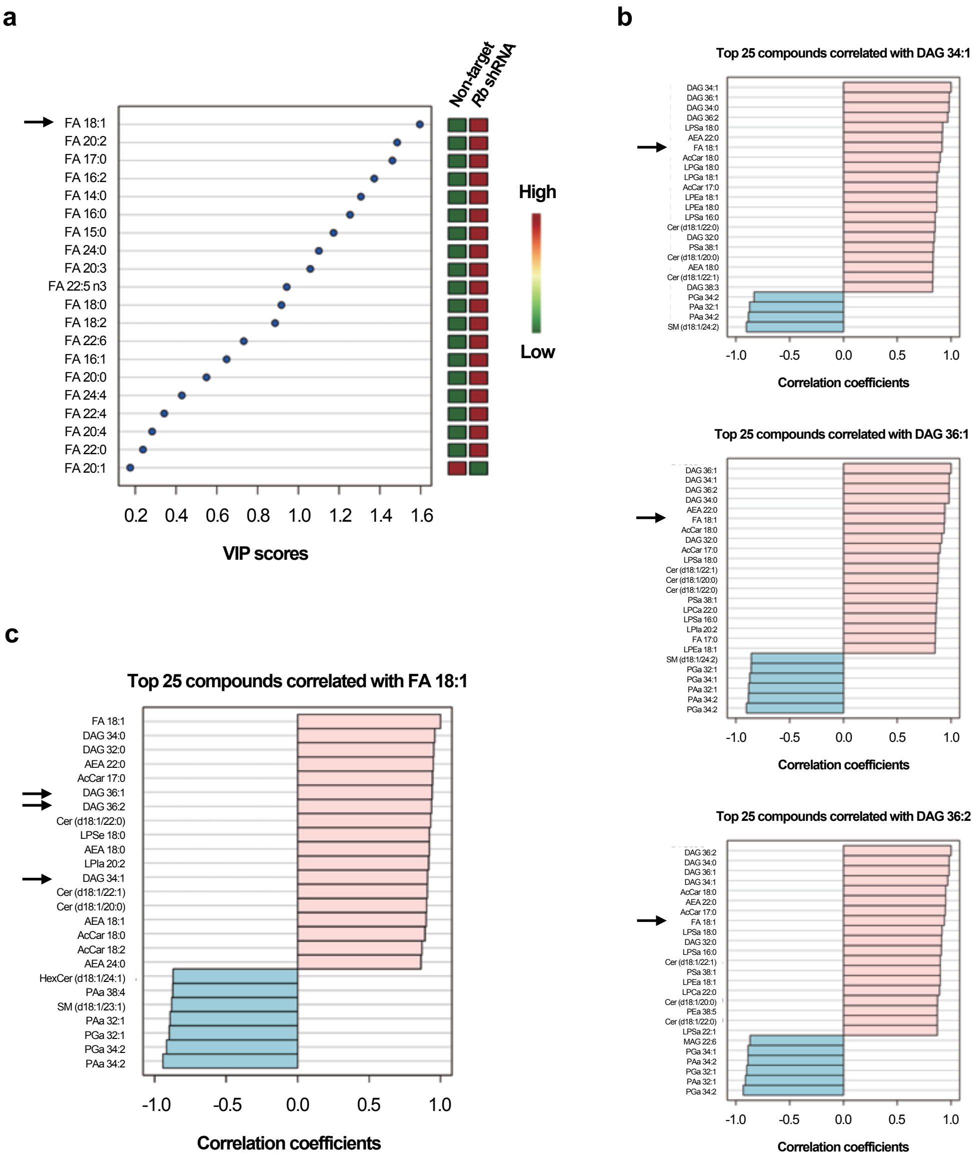

Muranaka et al, Supplementary Figure S5

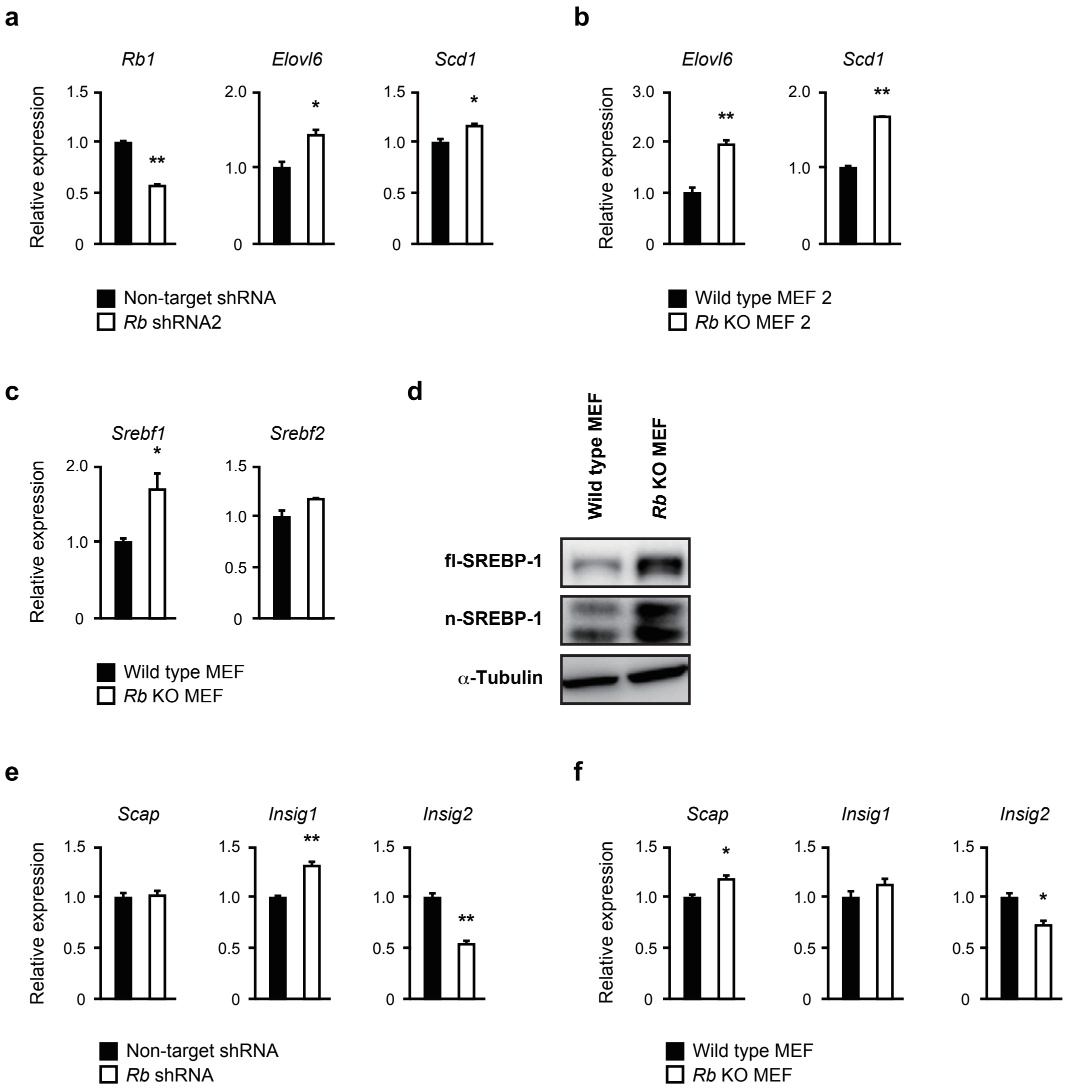

Muranaka et al, Supplementary Figure S6

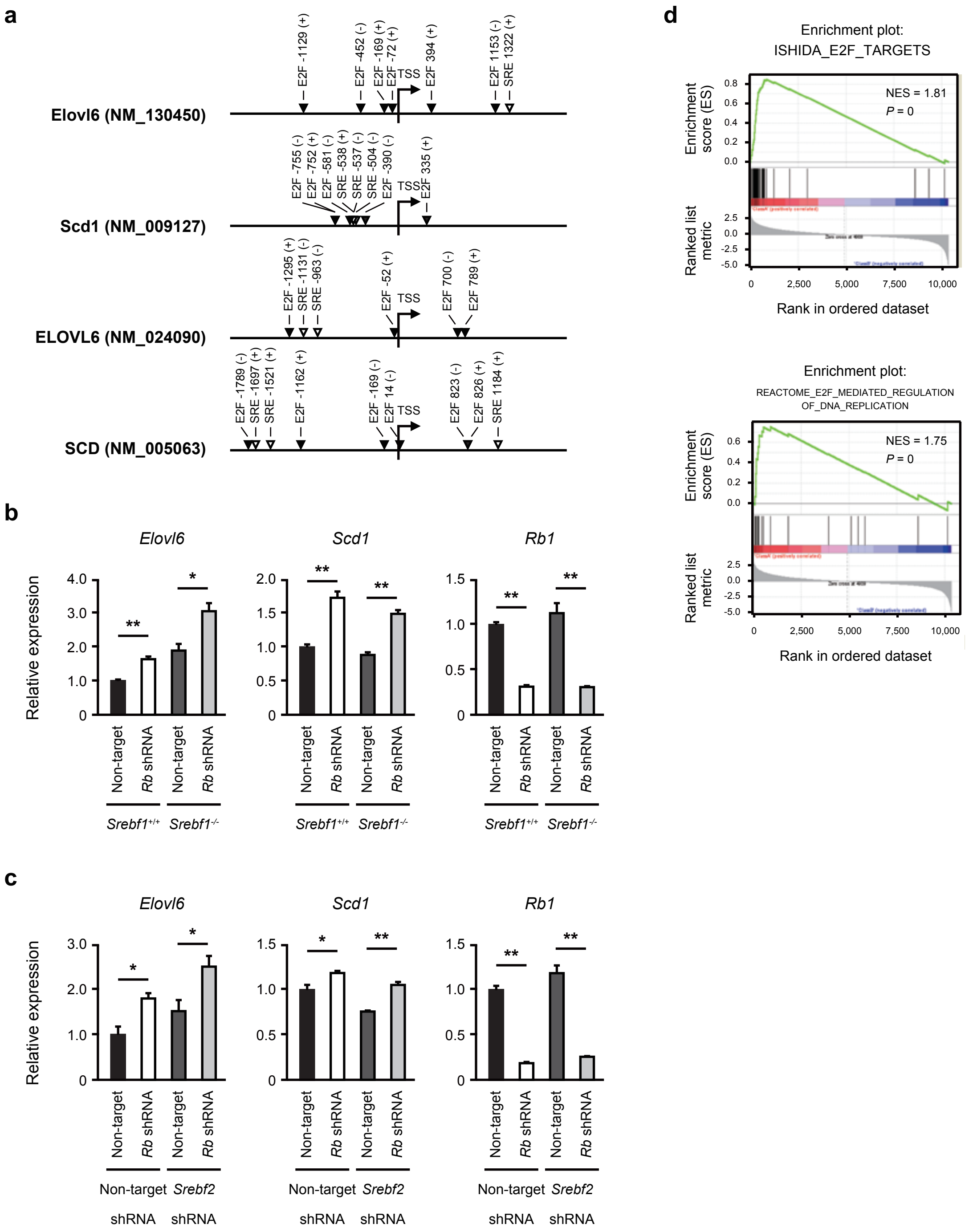

Muranaka et al, Supplementary Figure S7

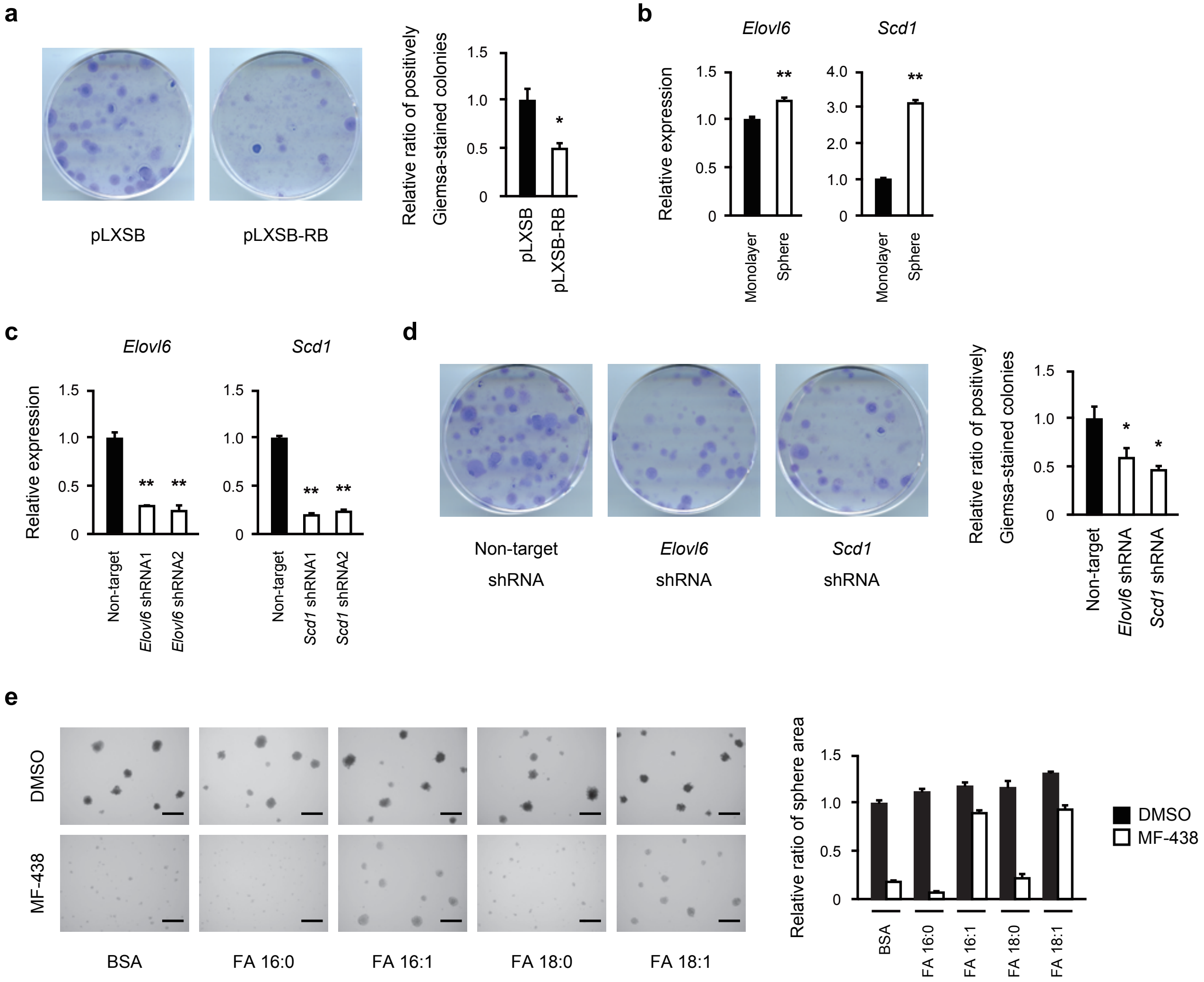

Muranaka et al, Supplementary Figure S8

a

Breast Cancer
(METABRIC, Nature 2012 & Nat Commun 2016)

ELOVL6

b

Ovarian Serous Cystadenocarcinoma
(TCGA, Nature 2011)

SCD1

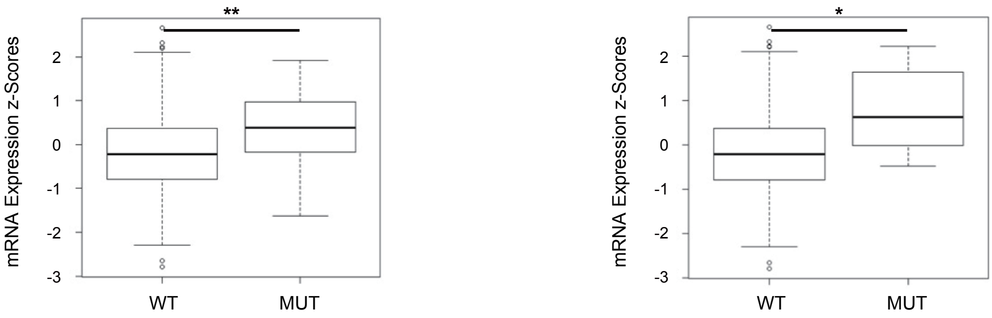

P = 0.006466

P = 0.01768
