# Supplementary material for: A distinct function of the retinoblastoma protein in the control of lipid composition identified by lipidomic profiling
Source: Oncogenesis. 2017 Jun 26;6(6):e350–. doi: 10.1038/oncsis.2017.51 (PMC5519198; doi:10.1038/oncsis.2017.51)
Supplement: Supplementary Table S5, S7-S10 [file oncsis201751x5.pdf]

Muranaka et al, Supplementary Table S5

a Up-regulated in *Rb*-depleted MEFs

KEGG Pathway Term	P-Value	Benjamini
DNA replication	9.9E-21	1.2E-18
Mismatch repair	2.0E-10	1.2E-8
Cell cycle	1.8E-7	7.3E-6
Homologous recombination	7.2E-7	2.2E-5
Nucleotide excision repair	3.1E-5	7.6E-4
Base excision repair	1.5E-4	3.0E-3
Basal cell carcinoma	1.1E-3	1.9E-2
Pathways in cancer	1.4E-3	2.1E-2
Vascular smooth muscle contraction	2.9E-3	3.8E-2
Pyrimidine metabolism	2.4E-2	2.5E-1

b Down-regulated in *Rb*-depleted MEFs

KEGG Pathway Term	P-Value	Benjamini
Cytokine-cytokine receptor interaction	6.6E-13	8.7E-11
Chemokine signaling pathway	3.6E-11	2.4E-9
NOD-like receptor signaling pathway	6.6E-11	2.9E-9
Graft-versus-host disease	1.8E-9	6.0E-8
Allograft rejection	1.6E-8	4.2E-7
Type I diabetes mellitus	5.3E-8	1.2E-6
Antigen processing and presentation	5.4E-8	1.0E-6
Toll-like receptor signaling pathway	2.1E-7	3.4E-6
Natural killer cell mediated cytotoxicity	2.5E-7	3.7E-6
Systemic lupus erythematosus	3.9E-7	5.1E-6

Muranaka et al, Supplementary Table S7

Symbol	Description	FC	P-Value
Acly	ATP citrate lyase	1.20	0.01
Acly	ATP citrate lyase	1.10	0.17
Acaca	acetyl-Coenzyme A carboxylase alpha	1.16	0.01
Acaca	acetyl-Coenzyme A carboxylase alpha	1.15	0.00
Acaca	acetyl-Coenzyme A carboxylase alpha	1.05	0.70
Acaca	acetyl-Coenzyme A carboxylase alpha	1.09	0.86
Fasn	fatty acid synthase	1.11	0.35
Elovl1	elongation of very long chain fatty acids (FEN1/Elo2, SUR4/Elo3, yeast)-like 1	1.15	0.05
Elovl1	elongation of very long chain fatty acids (FEN1/Elo2, SUR4/Elo3, yeast)-like 1	1.17	0.23
Elovl4	elongation of very long chain fatty acids (FEN1/Elo2, SUR4/Elo3, yeast)-like 4	1.19	0.07
Elovl4	elongation of very long chain fatty acids (FEN1/Elo2, SUR4/Elo3, yeast)-like 4	0.98	0.59
Elovl5	ELOVL family member 5, elongation of long chain fatty acids (yeast)	1.61	0.00
Elovl6	ELOVL family member 6, elongation of long chain fatty acids (yeast)	1.94	0.00
Elovl7	ELOVL family member 7, elongation of long chain fatty acids (yeast)	2.50	0.00
Scd3	stearoyl-coenzyme A desaturase 3	0.87	0.35
Scd1	stearoyl-coenzyme A desaturase 1	1.74	0.00
Scd3	stearoyl-coenzyme A desaturase 3	1.81	0.00
Scd2	stearoyl-coenzyme A desaturase 2	0.83	0.01

Muranaka et al, Supplementary Table S8

a Shamma A et al. Cancer Cell 2009; 15: 255-269

KEGG Pathway Term	P-Value	Benjamini
Steroid biosynthesis	1.4E-7	1.6E-5
Terpenoid backborne biosynthesis	5.2E-5	2.9E-3
p53 signaling pathway	2.3E-2	5.8E-1
Gap junction	4.7E-2	7.4E-1
Biosynthesis of unsaturated fatty acids	6.8E-2	7.9E-1

Symbol	Description
Elovl6	ELOVL family member 6, elongation of long chain fatty acids (yeast)
Scd2	stearoyl-Coenzyme A desaturase 2
Scd1	stearoyl-Coenzyme A desaturase 1

b Kitajima S et al. Oncogene 2017; in press

KEGG Pathway Term	P-Value	Benjamini
Cytokine-cytokine receptor interaction	1.5E-9	2.1E-7
Neuroactive ligand-receptor interaction	1.1E-6	7.6E-5
Hematopoietic cell lineage	3.0E-4	1.4E-2
Steroid biosynthesis	6.6E-4	2.3E-2
Complement and coaqulation cascades	1.6E-3	4.5E-2
Calcium signaling pathway	1.9E-3	4.4E-2
Biosynthesis of unsaturated fatty acids	6.0E-3	1.1E-1

Symbol	Description
Elovl2	elongation of very long chain fatty acids (FEN1/Elo2, SUR4/Elo3, yeast))-like 2
Fads2	fatty acid desaturase 2
Elovl6	ELOVL family member 6, elongation of long chain fatty acids (yeast)
Scd3	stearoyl-Coenzyme A desaturase 3
Scd4	stearoyl-Coenzyme A desaturase 4

c Markey MP et al. Oncogene 2007; 26: 6307-6318

KEGG Pathway Term	P-Value	Benjamini
DNA replication	7.7E-7	6.1E-5
Steroid biosynthesis	5.2E-4	2.0E-2
Mismatch repair	1.1E-3	2.9E-2
Cell cycle	1.3E-3	2.5E-2
Nucleotide excision repair	7.8E-3	1.2E-1
Pyrimidine metabolism	1.3E-2	1.6E-1
Biosynthesis of unsaturated fatty acids	2.7E-2	2.7E-1

Symbol	Description
Fads1	fatty acid desaturase 1
Elovl6	ELOVL family member 6, elongation of long chain fatty acids (yeast)
Scd1	stearoyl-Coenzyme A desaturase 1

Muranaka et al, Supplementary Table S9

Elovl6 (NM_130450)

ID	Score	Loc.	Str.	Sequence
V$E2F_Q6	0.861	-1129	(+)	TGGCCCGAACCTT
V$E2F_02	0.872	-452	(-)	GCGCCCAA
V$E2F_02	0.872	-169	(+)	CTTGGCGC
V$E2F_02	0.872	-72	(+)	TCTCGCGC
V$E2F_02	0.874	394	(+)	TTTCGCGA
V$E2F_02	0.893	1153	(-)	CCGGGAAA
V$SREBP1_02	0.907	1322	(+)	GATCTCCCCAC

Scd1 (NM_009127)

ID	Score	Loc.	Str.	Sequence
V$E2F_Q6	0.851	-755	(-)	TGTTTTCCCGGGT
V$E2F_02	0.893	-752	(+)	TTTCCCGG
V$E2F_02	0.872	-581	(-)	GCGCCAGA
V$SREBP1_01	0.859	-538	(+)	AATCACGTTAT
V$SREBP1_01	0.883	-537	(-)	ATCACGTTATC
V$SREBP1_02	0.886	-504	(+)	CATCAGCCCAC
V$E2F_02	0.872	-390	(-)	GCACCAAA
V$E2F_02	0.872	335	(+)	TTTCGTGC

ELOVL6 (NM_001130721)

ID	Score	Loc.	Str.	Sequence
V$E2F_02	0.872	-1344	(+)	TTTGGCTC
V$SREBP1_01	0.948	-1180	(-)	CTCAGGTGATC
V$SREBP1_02	0.886	-1012	(-)	GTGGGGGGATG
V$E2F_02	0.872	-101	(+)	TTTCGCAC
V$E2F_02	0.872	651	(-)	GCGCGACA
V$E2F_02	0.877	740	(+)	TTTCTCGC

ELOVL6 (NM_024090)

ID	Score	Loc.	Str.	Sequence
V$E2F_02	0.872	-1295	(+)	TTTGGCTC
V$SREBP1_01	0.948	-1131	(-)	CTCAGGTGATC
V$SREBP1_02	0.886	-963	(-)	GTGGGGGGATG
V$E2F_02	0.872	-52	(+)	TTTCGCAC
V$E2F_02	0.872	700	(-)	GCGCGACA
V$E2F_02	0.877	789	(+)	TTTCTCGC

SCD (NM_005063)

ID	Score	Loc.	Str.	Sequence
V$E2F_02	0.872	-1789	(-)	GCTCGAAA
V$SREBP1_01	0.948	-1697	(+)	GATCACCTGAG
V$SREBP1_02	0.907	-1521	(+)	GATCACACCAC
V$E2F_02	0.872	-1162	(+)	ATTGGCGC
V$E2F_02	0.872	-169	(-)	GCCCCAAA
V$E2F_02	0.872	14	(-)	GCACCAAA
V$E2F_Q6	0.941	823	(-)	AGGTTTCGCGCCC
V$E2F_02	1.000	826	(+)	TTTCGCGC
V$SREBP1_02	0.907	1184	(+)	TATGACCCCAC

Muranaka et al, Supplementary Table S10

Lipid Class	Lipid Class	ion mode	Q1:Parent Ion	Q3:Daughter Ion	CE
Sphingosine−1−phosphate (d18:1)	S1P	negative	M−H	79[PO3]	−50
Sphingosine (d18:1)	Sph	negative	M−H+CH3COOH	59[CH3COO]	−28
Phosphatidylcoline	PC	negative	M+CH3COO	Q1−74[Me+CH3COO]	−30
Phosphatidylethanolamine	PE	negative	M−H	196[GroPtdEtn−H2O]	−60
Sphingomyerine	SM	negative	M+CH3COO	Q1−74[Me+CH3COO]	−40
Ceramide	Cer	negative	M−H+CH3COOH	Q1−60[CH3COOH]	−45
Glucosylceramide, Galactosylceramide	HexCer	negative	M−H+CH3COOH	Q1−222[CH3COOH+Hxose−H2O]	−60
Phosphatidylserine	PS	negative	M−H	Q1−87[Ser]	−40
Phosphatidylinositol	PI	negative	M−H	241[PtdIns−H2O]	−60
Phosphatidic acid	PA	negative	M−H	153[GroPtd−H2O]	−50
LPhosphatidylcoline	LPC	negative	M+CH3COO	Q1−74[Me+CH3COO]	−30
LPhosphatidylethanolamine	LPE	negative	M−H	196[GroPtdEtn−H2O]	−40
LPhosphatidylserine	LPS	negative	M−H	Q1−87[Ser]	−30
LPhosphatidylinositol	LPI	negative	M−H	241[PtdIns−H2O]	−45
LPhosphatidylglycerol	LPG	negative	M−H	Q1−228[GroPtdGro−H2O]	−35
LPhosphatidic acid	LPA	negative	M−H	153[GroPtd−H2O]	−35
Fatty acid (number of double bonds: 0−3)	FA	negative	M−H	Q1−18[H2O]	−32
Fatty acid (number of double bonds: 4−8)	FA	negative	M−H	Q1−44[CO2]	−29
Phosphatidylglycerol	PG	positive	M+H+NH3	Q1−189[PtdGro+NH3]	40
Diacylglycerol	DAG	positive	M+H+NH3	Q1−35[H2O+NH3]	31
Monoacylglycerol	MAG	positive	M+H+NH3	Q1−109[Gro+NH3]	15
Acylethanolamide	AEA	positive	M+H	62[Etn]	36
Acylcarnitine	AcCar	positive	M+H	85.1	32

Gro: glycerol

Ptd: Phosphatidyl

Etn: Ethanolamine

Ins: Inositol
