# Supplementary material for: A distinct function of the retinoblastoma protein in the control of lipid composition identified by lipidomic profiling
Source: Oncogenesis. 2017 Jun 26;6(6):e350–. doi: 10.1038/oncsis.2017.51 (PMC5519198; doi:10.1038/oncsis.2017.51)
Supplement: Supplementary Figure Legends and Tabels [file oncsis201751x6.docx]

MS#ONCSIS-17-0052-T-R

**Supplementary Figure Legends and Tables**

**A distinct function of the retinoblastoma protein in the control of lipid composition identified by lipidomic profiling**

**Hayato Muranaka^1^, Akio Hayashi^2^, Keiichi Minami^2^, Shunsuke Kitajima^1,3^, Susumu Kohno^1^, Yuuki Nishimoto^1^, Naoko Nagatani^1^, Misa Suzuki^1^, Nilakshi Kulathunga^1^, Nobunari Sasaki^1,4^, Nobuhiro Okada^1^, Takashi Matsuzaka^5^, Hitoshi Shimano^5^, Hideaki Tada^2^, Chiaki Takahashi^1,*^**

Supplementary Figure S1. Lipidomic profiling of *Rb*-depleted MEFs.

(a) Cell proliferation of the indicated MEFs. Wild type MEFs were infected with lentiviruses expressing the indicated shRNAs and selected. Cells were seeded into 100 mm dishes and cultured in the medium containing 1% FBS for 24 hours. Data represent mean + S.D. (n=4) * *P* < 0.05, ** *P* < 0.01 by Student’s *t*-test.

(b) Representative mass spectra of control MEFs.

(c) PLS-DA score plot of 199 lipids (n=4) in *Rb*-depleted MEFs as compared to control MEFs.

(d) Hierarchical clustering analysis for lipids with top 25 VIP scores.

(e) Levels of the indicated lipids in control MEFs (solid) and *Rb*-depleted MEFs (blank). Data represent mean + S.D. (n=4) * *P* < 0.05, ** *P* < 0.01 by Student’s *t*-test.

Supplementary Figure S2. The impact of Rb loss on DAG composition.

(a) Top VIP scores with heat map from PLS-DA of DAG species. Red and green indicate increased and decreased levels, respectively.

(b) Hierarchical clustering analysis for DAG species.

Supplementary Figure S3. The impact of Rb loss on lipid composition.

(a-h) Heat map represents log 2 fold changes in the indicated lipid species relative to control MEFs. Red, increase; white, average; blue, decrease.

Supplementary Figure S4. The impact of Rb loss on FA composition.

(a) Top VIP scores and heat map from PLS-DA of FAs. Red and green indicate increased and decreased levels, respectively.

(b) Correlation analysis was used to identify which features are correlated with DAG 34:1, DAG 36:1, and DAG 36:2. Top 25 lipids correlated with the indicated DAG species are shown. Red, positive correlation; blue, negative correlation. FA 18:1 is indicated by an arrow.

(c) Correlation analysis to identify the features correlated with FA 18:1. Top25 lipids correlated with FA 18:1 are shown. Red, positive correlation; blue, negative correlation. DAG species possibly composed of C18:1 acyl chains are indicated by arrow.

Supplementary Figure S5. The impact of Rb status on SREBP-1 nuclear translocation.

(a) RT-qPCR of the indicated genes in wild type MEFs infected with lentiviruses expressing the indicated shRNAs and selected. Cells were cultured in the medium containing 1% FBS for 24 hours. Data represent mean + S.D. (n=3) * *P* < 0.05, ** *P* < 0.01 by Student’s *t*-test.

(b) RT-qPCR of the indicated genes in *Rb*^+/+^ MEFs and *Rb*^-/-^ MEFs (different batches of MEFs from those used in Figure 4e). Cells were cultured in the medium containing 1% FBS for 24 hours. Data represent mean + S.D. (n=3) * *P* < 0.05, ** *P* < 0.01 by Student’s *t*-test.

(c) RT-qPCR of indicated genes in *Rb*^+/+^ MEFs and *Rb*^-/-^ MEFs. Cells were cultured in the medium containing 1% FBS for 24 hours. Data represent mean + S.D. (n=3) * *P* < 0.05, ** *P* < 0.01 by Student’s *t*-test.

(d) IB of the indicated proteins in *Rb*^+/+^ MEFs and *Rb*^-/-^ MEFs. Cells were cultured in the medium containing 1% FBS for 24 hours.

(e) RT-qPCR of the indicated genes in wild type MEFs infected with lentiviruses expressing the indicated shRNAs and selected. Cells were cultured in the medium containing 1% FBS for 24 hours. Data represent mean + S.D. (n=4) * *P* < 0.05, ** *P* < 0.01 by Student’s *t*-test.

(f) RT-qPCR of indicated genes in *Rb*^+/+^ MEFs and *Rb*^-/-^ MEFs. Cells were cultured in the medium containing 1% FBS for 24 hours. Data represent mean + S.D. (n=3) * *P* < 0.05, ** *P* < 0.01 by Student’s *t*-test.

Supplementary Figure S6. The involvement of SREBPs in the regulation of *Elovl6* and *Scd1* gene expression by Rb.

(a) E2F- and SREBP- binding consensus sequences appeared on the promoter of the indicated genes. Schematic presentation of promoter structure of the indicated genes in mouse and human genome. Positions of the DNA binding consensus sequence for E2Fs and SREBPs are indicated.

(b) RT-qPCR of the indicated genes in MEFs with the indicated genotypes infected with lentiviruses expressing the indicated shRNAs and selected. Cells were cultured in the medium containing 1% FBS for 24 hours. Data represent mean + S.D. (n=3) * *P* < 0.05, ** *P* < 0.01 by Student’s *t*-test.

(c) RT-qPCR of the indicated genes in wild type MEFs infected with lentiviruses expressing the indicated shRNAs and selected. Cells were cultured in the medium containing 1%FBS for 24 hours. Data represent mean + S.D. (n=3) * *P* < 0.05, ** *P* < 0.01 by Student’s *t*-test.

(d) GSEA results for E2F target gene set in *Rb*-depleted MEFs vs. control MEFs.

Supplementary Figure S7. The effect of *Elovl6* and *Scd1* depletion on colony formation and tumorigenicity in RN6 cells.

(a) Colony formation assay of RN6 cells infected with pLXSB or pLXSB-RB and selected. 1 X 10^3^ cells were seeded into 60 mm dishes and after 10 days of culture, colonies were stained with Giemsa solution. Data represent mean + S.D. (n=3) * *P* < 0.05, ** *P* < 0.01 by Student’s *t*-test.

(b) RT-qPCR of the indicated genes in RN6 cells cultured under the indicated conditions. Data represent mean + S.D. (n=3-5). * *P* < 0.05, ** *P* < 0.01 by Student’s *t*-test.

(c) RT-qPCR of the indicated genes in RN6 cells infected with lentiviruses expressing the indicated shRNAs and selected. Data represent mean + S.D. (n=3). * *P* < 0.05, ** *P* < 0.01 by Student’s *t*-test.

(d) Colony formation assay of RN6 cells infected with lentiviruses expressing the indicated shRNAs and selected. 1 X 10^3^ cells were seeded into 60 mm dishes and after 10 days of culture, colonies were stained with Giemsa solution. Data represent mean + S.D. (n=3) * *P* < 0.05, ** *P* < 0.01 by Student’s *t*-test.

(e) Sphere assay of RN6 cells treated with MF-438 (10 μM) and indicated BSA-conjugated fatty acids (10 μM). 2 X 10^3^ cells were seeded and spheres images were acquired after 7 days. Scale bars, 300 μm. Data represent mean + S.D. (n=4).

Supplementary Figure S8. Correlation of *RB* mutation with *ELOVL6* and *SCD1* gene expression in human cancer patients.

(a-b) Expression data for *ELOVL6* in breast cancer (n=1,866, METABRIC, Nature, 2012, Nat. Commun 2016) and *SCD1* in ovarian serous cystadenocarcinoma (n=316, TCGA, Nature, 2011) were obtained from cBioPortal (http://www.cbioportal.org/). Student's *t*-test was performed to assess the significance of the increases in expression levels for *RB* mutated samples to those with wild type. * *P* < 0.05, ** *P* < 0.01 by Student’s *t*-test.

Supplementary Table S1. 199 lipids detected in *Rb*-depleted MEFs and control MEFs by LC-MS/MS.

Supplementary Table S2. Lipids with the VIP values of > 1.0 between *Rb*-depleted MEFs and control MEFs.

Supplementary Table S3. Fold change in each DAG species.

Supplementary Table S4. Gene expression in *Rb*-depleted MEFs and control MEFs analyzed by microarray. Genes with both maximum *p* value of 0.01 and minimum fold change of 2.0 were selected.

Supplementary Table S5. Pathway analysis in microarray data of *Rb*-depleted MEFs and control MEFs by DAVID. (a) Up-regulated in *Rb*-depleted MEFs. (b) Down-regulated in *Rb*-depleted MEFs.

Supplementary Table S6. Fold change and *p* value in expression of lipid metabolism genes in microarray data of *Rb*-depleted MEFs and control MEFs.

Supplementary Table S7. Fold change and *p* value in expression of fatty acid synthesis genes in microarray data of *Rb*-depleted MEFs and control MEFs.

Supplementary Table S8. Pathway analysis in other gene expression data sets by DAVID. (a) Shamma et al. 2009, (b) Kitajima et al. 2017, (c) Markey et al. 2007

Supplementary Table S9. E2F- and SREBP-binding sequences found in the promoter of mouse *Elovl6* and *Scd1*, and human *ELOVL6* and *SCD1*.

Supplementary Table S10. Internal standards for lipidomics analysis used in this study.
