# Supplementary material for: A distinct function of the retinoblastoma protein in the control of lipid composition identified by lipidomic profiling
Source: Oncogenesis. 2017 Jun 26;6(6):e350–. doi: 10.1038/oncsis.2017.51 (PMC5519198; doi:10.1038/oncsis.2017.51)
Supplement: Supplementary Materials and Methods [file oncsis201751x7.docx]

MS#ONCSIS-17-0052-T-R

**Supplementary Materials and Methods**

**A distinct function of the retinoblastoma protein in the control of lipid composition identified by lipidomic profiling**

**Hayato Muranaka^1^, Akio Hayashi^2^, Keiichi Minami^2^, Shunsuke Kitajima^1,3^, Susumu Kohno^1^, Yuuki Nishimoto^1^, Naoko Nagatani^1^, Misa Suzuki^1^, Nilakshi Kulathunga^1^, Nobunari Sasaki^1,4^, Nobuhiro Okada^1^, Takashi Matsuzaka^5^, Hitoshi Shimano^5^, Hideaki Tada^2^, Chiaki Takahashi^1,*^**

Reagents

The following reagents were used: SREBP inhibitor Fatostatin (#341329), E2F inhibitor HLM006474 (#324461) and SCD1 inhibitor MF-438 (#569406) were purchased from Calbiochem. BSA-conjugated oleic acid (#O3008), fatty acid-free bovine serum albumin (BSA, #A8806), Stearic acid (#S4751), Palmitic acid (#P5585) and Palmitoleic acid (#P9417) were purchased from Sigma-Aldrich.

Lentiviral production

MISSION TRC shRNA target sets used in this study were as follows: mouse *Rb1* (TRCN0000042543 and TRCN0000042544), mouse *Srebf2* (TRCN0000176022), mouse *Elovl6* (TRCN0000175486 and TRCN0000173809), mouse *Scd1* (TRCN0000114331 and TRCN0000114332) and negative control (Non-target; SHC002).

Lipidomics

LC-MS/MS was performed using Acquity UPLC system coupled with 4000QTRAP triple quadrupole mass spectrometer (AB SCIEX) equipped with electrospray ionisation (ESI) source. UPLC separation was achieved using Acquity BEH-C18 reverse-phase column for UPLC system (1.7 μm particle size, 1.0 mm i.d. X100 mm) at 50℃, sample injection volume was 4 μL at a flow rate of 0.080 mL/min. The mobile phase A consists of ultra pure water with 0.5 mM CH3COONH4 and 0.1 mM H3PO4, and B of acetonitrile/methanol (4:1). The mobile phase kept initially at 27% B for 1.0 min, the gradient started at 27% B increased linearly to 66% B in 2.0 min, from 66% B to 85% B in 10.0 min, from 85% B to 100% B in 6.0 min, then kept at 100% B for 13.0 min and finally back to initial phase. Every lipid species were monitored by sMRM. Selected parent ion (Q1), daughter ion (Q3) and collision energy (CE) are shown in Supplementary Table S9. Conditions of sMRM analysis included a CUR of 10, a GS1 of 50, a GS2 of 50 for negative ion mode and of 30 for positive, IS of -4,500 V for negative and 5,500 V for positive. Following are internal standards used for lipidomics: C8 Glucosyl (ß) Ceramide (d18:1/8:0), 1,3-18:1 D5 DG, 17:0-LPA, 17:1-LPC, 17:1-LPE, 17:1-LPG, 17:1-LPI, 17:1-LPS, 8.9-MAG (17:1-MAG), 16:0 D31-18:1 PA, 16:0 D31-18:1 PC, 16:0 D31-18:1 PE, 16:0-18:1 D5 PG, 16:0 D31-18:1 PI, 16:0 D31-18:1 PS, Sphingosine (d20:1), SM (d18:1/6:0) and Cer (d17:1/18:0) were purchased from Avanti Polar Lipids. Isovalerylcarnitine (Car 5:0) was purchased from Larodan Fine Chemicals. Palmitic acid-d4 was purchased from Wako Pure Chemicals. Oleoyl Ethanolamide-d4 and Arachidonic acid-d8 were purchased from Cayman Chemical. For more detailed information on internal standards, see Supplementary Table S10.

Microarray analysis

Total RNA was extracted using the RNeasy Mini Kit (#74106, Qiagen) according to the manufacturer’s instructions. To determine the quality of RNA, RNA Integrity Number (RIN) was used by Agilent 2100 Bioanalyzer. The microarray analysis was performed with SurePrint G3 Mouse Gene Expression 8X60K Microarray Kit (#G4852A, Agilent Technologies). Data were analyzed by Expressionist Analyst software version 8.2.7b (Genedata). The raw data are available in Gene Expression Omnibus (GEO) database (GSE90571). Pathway analysis was performed on GO analysis and KEGG analysis systems provided by DAVID v6.7 (https://david.ncifcrf.gov/). GSEA (<http://www.broadinstitute.org/gsea/index.jsp>) was performed on Signal-to-Noise metrics using gene sets obtained from “C2 all v5.1” gene sets.

RT-qPCR

TaqMan probes used in this study were as follows: *Acaca* (Mm01304257_m1), *Acly* (Mm01302282_m1), *Actb* (Mm02619580_g1), *Elovl6* (Mm00851223_s1), *Fasn* (Mm00662319_m1), *Hprt* (Mm01545399_m1), *Insig1* (Mm00463389_m1), *Insig2* (Mm00460121_m1), *Rb1* (Mm00485586_m1), *RB1* (Hs01078066_m1), *Scap* (Mm01250176_m1), *Scd1* (Mm00772290_m1), *Srebf1* (Mm00550338_m1), *Srebf2* (Mm01306292_m1).

Immunoblotting

Antibodies used in this study were as follows: SREBP-1 (sc-13551, Santa Cruz Biotechnology), α-Tubulin (CP06, Calbiochem), FASN (#3180, Cell Signaling Technology), SCD (#2438, Cell Signaling Technology) and RB (#554136, BD Pahrmingen).

Cross-linked chromatin immunoprecipitation (ChIP)

MEFs (80% confluent in 100 mm dish) were cross-linked with 10 ml 1% formaldehyde in medium for 10 min at room temperature and incubated in 10 ml 125 mM glycine in medium for 5 min to quench reactive aldehydes.Cells were harvested using a cell scraper and rinsed twice with cold-PBS, immersed in 1 ml lysis buffer (50 mM Tris-HCl [pH 7.5], 150 mM NaCl, 5 mM EDTA, 0.5% NP-40, 1.0% Triton X-100 and protease inhibitor cocktail) at 4°C for 10 min. After centrifugation, cells were resuspended in 100 μl SDS lysis buffer (50 mM Tris-HCl [pH 8.0], 10 mM EDTA, and 1% SDS), 360 μl ChIP dilution buffer (50 mM Tris-HCl [pH 8.0], 167 mM NaCl, 1.1% Triton X-100, 0.11% sodium deoxycholate), 40 μl 10% SDS and protease inhibitor cocktail. Cells were sonicated using Qsonica Q125 (WAKEN). After centrifugation, 4.5 ml ChIP dilution buffer with protease inhibitor cocktail were added to the supernatant to yield the input for ChIP. The size of DNA fragment was determined by agarose gel electrophoresis as ~300–1500 bp (median ~500 bp). For cross-linked ChIP, an aliquot of ChIP input (1 ml) was incubated with 2 μg anti-E2F1 (sc-193, Santa Cruz Biotechnology), 2 μg anti-E2F2 (sc-633, Santa Cruz Biotechnology), 2 μg anti-E2F3 (sc-878, Santa Cruz Biotechnology) or 2 μg Rabbit IgG (148-09551, Wako) overnight at 4°C with rotation. 50 μl original suspension of Dynabeads Protein G (DB10004, Invitrogen) were washed three times with 1 ml RIPA-150 mM NaCl (50 mM Tris-HCl [pH 8.0], 150 mM NaCl, 1 mM EDTA, 0.1% SDS, 1% Triton X-100, 0.1% sodium deoxycholate) and incubated with 1 ml ChIP input at 4°C for 3 h with rotation. Beads were then washed sequentially with 1 ml RIPA-150 mM NaCl, 1 ml RIPA-500 mM NaCl, 1 ml LiCl buffer (10 mM Tris-HCl [pH 8.0], 0.25 M LiCl, 1 mM EDTA, 0.5% NP-40, 0.5% sodium deoxycholate) and twice with 1 ml TE (10 mM Tris-HCl [pH 8.0] and 1 mM EDTA). After removing TE, beads were mixed with 200 μl elution buffer (10 mM Tris-HCl [pH 8.0], 300 mM NaCl, 5 mM EDTA, and 0.5% SDS) and incubated overnight at 65°C to reverse cross-linking. Samples were then treated with 2 μl 0.5 mg/ml RNase (312-01931, Wako) at 37°C for 1 h and 5 μl 20 mg/ml Proteinase K (9033, Takara) at 55°C for 3 h. DNA was extracted by QIAquick PCR Purification Kit (28106, QIAGEN) and finally eluted with 30 μl Buffer EB. Real-time PCR was performed for each sample (1.0 μl equivalent) with FastSYBR^TM^ Green Master Mix (4385612, Applied Biosystems) using Roche Light Cycler 480 II. Results were presented as percentages of input DNA. Primers used for ChIP-qPCR were as follows: Cdc6 F, 5’-GCGTGGTGATGAGTGACAAC-3’; Cdc6 R, 5’-AGCCTCACCTCTCTGGACAC-3’; Elovl6 promoter1 F, 5’-AGTCCACCAACCACCTTCTTG-3’; Elovl6 promoter1 R, 5’-CGTGCGATCGGGAAAATTAACC-3’; Elovl6 promoter2 F, 5’-AGTATGACCTCAAGGCCACTTCC -3’; Elovl6 promoter2 R, 5’-AATTAACCCTTTGCGCGAGAGG-3’; Fscn2 F, 5’-CATCCAGGAGCCACTGAAAT-3’; Fscn2 R, 5’-GACATGGACGCTACCTGCTC-3’; Scd1 promoter1 F, 5’-GCAGAGGGAACAGCAGATTGC-3’; Scd1 promoter1 R, 5’-TGTGAAGCCCGTCTTGTCATTG-3’; Scd1 promoter2 F, 5’-GTCTTCCTCACTTCTTTCGATGCG-3’; Scd1 promoter2 R, 5’-TGTGTGCAAGCCTAGGACTTTG-3’.

Promoter analysis

2 kb upstream and 2 kb downstream of the predicted transcription start site (TSS) of genes in genomic DNA sequences were obtained from DBTSS (http://dbtss_old.hgc.jp/hg19_mm9/). The sequences were searched for the sterol responsive element (SRE) and E2F binding consequences by TFBIND (<http://tfbind.hgc.jp/>).

Patient data

Expression data for *ELOVL6* in breast cancer (n=1,866, METABRIC, Nature, 2012, Nat. Commun 2016) and *SCD1* in ovarian serous cystadenocarcinoma (n=316, TCGA, Nature, 2011) were obtained from cBioPortal (http://www.cbioportal.org/). Student's *t*-test was performed to assess the significance of the increases in expression levels for RB mutated samples to those with wild type.
